# Stakeholders’ perceptions of protected area management following a nationwide community-based conservation reform

**DOI:** 10.1371/journal.pone.0215437

**Published:** 2019-04-24

**Authors:** Sigrid Engen, Per Fauchald, Vera Hausner

**Affiliations:** 1 Department of Arctic and Marine Biology, UIT-The Arctic University of Norway, Tromsø, Norway; 2 Norwegian Institute for Nature Research, Tromsø, Norway; Wildlife Conservation Society Canada, CANADA

## Abstract

People’s perceptions can point to reasons for conservation support or lack thereof. In this study, we surveyed the perceptions of conservation among local stakeholders who participated in protected area (PA) management following a reform towards community-based conservation in Norway. We analyzed the link between perceptions of threats to conservation values, prioritized management actions and trust in PA governance, and assessed how these perceptions aligned with the stakeholders’ preferred overarching conservation approach and their support for PAs. Conservation perceptions differed mostly between property owners and representatives of nature conservation (such as NGOs). Property owners perceived modern farming, grazing and hay making, and securing the interests of rights holders as a priority. They had a lower support for PAs and favored a conservation approach focusing on “people and nature”. Representatives from nature conservation prioritized management actions to increase biodiversity and reduce land development, had higher trust in environmental authorities and identified motorized vehicle use as a threat to conservation values. They had a high support for PAs and favored a conservation approach that mitigates threats from human activity (i.e. “nature despite people”). The nationwide reform aimed at increasing support for PAs, but 31% of the members of the stakeholder advisory councils were willing to downgrade or degazette PAs for the benefit of economic development, which is much more than general population surveys. However, the level of trust in local governance was less polarized among the members of stakeholder advisory councils compared with the former state governance, which suggests that that the community-based conservation reform has the potential to improve collaboration and conflict mitigation.

## 1. Introduction

Community-based conservation that places decision-making power with lower-level authorities and involves local stakeholders in conservation management, is thought to increase support for conservation through greater sensitivity to local conditions and perceptions [1–3]. Community-based conservation is loosely defined as ‘‘natural resources or biodiversity protection by, for, and with the local community” [4] and includes acts of decentralization i.e., when the government grants decision making power to local governing bodies [5]. Community-based conservation is characterized by a bottom-up process where decision making starts at the local level and involves interactions at multiple levels [6]. Closeness to those affected by conservation is believed to increase decision makers’ understanding of local conditions and needs, which in turn can increase trust and improve perceptions of- and support for conservation [7–11].

In 2009, the Norwegian government decided to implement a nation-wide community-based conservation strategy for protected areas. This shift in governance entailed a transfer of management authority from the government to local conservation boards [12–14] and these conservation boards are obligated to consult with local stakeholders [15]. The community-based conservation reform was the result of years of conflicts between national conservation agencies and local stakeholders [14,16–18] and the rationale behind the reform was to reduce tension by creating a sense of ownership to the protected areas [15]. Prior to the reform, reports had recurrently pointed out that conservation values (i.e., the reasons for protecting the areas, such as biodiversity, threatened species or habitats, science, recreation or cultural heritage) in protected areas were threatened [19,20], which was another reason for the change in governance [15].

People’s perception of the conservation problem is central to their attitudes to different conservation strategies [21–28]. For instance, people living in or next to protected areas in three European countries differed in their views of nature, such as whether nature is fragile or resilient, stable or dynamic and whether nature and culture are related or opposites [25,26]. Viewing ideal nature as untouched was associated with preferences for stricter regulations and exclusion whereas viewing nature as a product of people-nature interactions was associated with the need for land management [25]. Similarly, the view of nature, its use and ability to regenerate was a core issue in the long-standing conflict over a North-American national park (Voyageurs National Park, Minnesota), along with opinions about who should have management responsibility (i.e., local and state control or federal) [23]. These aspects are also central to conservation conflicts in Norwegian protected areas [24,29].

Support for protecting land is more likely if the way conservation is conceptualized and communicated by decision-makers and practitioners resonates with people (i.e., makes conservation seem natural and familiar) [30,31]. Four main approaches to conservation (“nature for itself”, “nature despite people”, “nature for people” and “people and nature”) have developed over time, according to Mace [32], all of which are in use today. These approaches are likely to resonate differently among local stakeholders, as they do among conservation scientists and practitioners [33–35].

PAs started as (so-called) nature-centered conservation and were initially conceptualized as islands of wilderness protected from human interventions [36]. This is the key conservation strategy within the “nature by itself” approach that dominated conservation thinking prior to the 1960s [32]. In the 1970s-1980s the “nature despite people” approach emerged from the realization of the increasing environmental impacts of human activity. With this approach focus shifted towards reversing or reducing threats to species and habitats from humans through population monitoring and management [32]. With the “nature despite people” approach the idea of PAs broadened to include PA networks (emphasizing ecological representation and the facilitation of species movement between PAs) and PAs in relation to the landscape, focusing on how buffer areas affect conservation values inside PAs [36].

More recently approaches that, instead of seeing humans as a threat to nature, recognize that people are a part of nature have emerged. The “nature for people” approach focuses on how ecosystems are essential for human welfare through the goods and services provided by nature. An ecosystem services approach to PAs could generate support for PAs through a greater understanding of the many different types of benefits provided by these conservation areas [37,38] and by focusing on the value of these benefits [39]. For example, local benefits generated from nature-based tourism in protected areas could potentially increase support for conservation. The “people and nature” approach also brings in people as an integral part of nature, but here conservation is perceived as managing social-ecological systems where people, culture and institutions are connected to nature through their use, modification and care for nature [40]. A social-ecological approach looks at PAs as land use embedded in multifunctional landscapes [41]. It incorporates social science in conservation planning alongside ecological knowledge [42] to better understand the complexity of human-nature dynamics, with the goal of developing policy that accounts for these dynamics [36,41–43].

By understanding perceptions (i.e. the way people observe, understand, interpret, and evaluate conservation) we can gain knowledge of the reasons for local support or resistance to environmental governance and management [9,44,45]. The purpose of our study was to investigate the perceptions of conservation among local stakeholders involved in protected area management following the nationwide community-based conservation reform. A recent review of existing knowledge of attitudes towards protected areas in Norway shows that while support for protection is high in the general population, less is known about the local attitudes and perceptions of conservation [46].

In this study, stakeholders’ perception of the conservation problem was operationalized as perceived threats to conservation values, their prioritized management actions and trust in the actors involved in PA governance. First, we asked: what are the relationships between stakeholders’ perceived threats, management priorities and trust in PA governance? Secondly, how are these perceptions related with stakeholders’ preferences for overarching conservation approaches (following Mace [32]) and their level of support for protected areas (based on their attitudes towards PA loss or degradation for economic activity). Finally, we discuss the implications for PA management following the 2009 community-based conservation reform.

### 1.1 The nationwide community-based management reform

Conservation has been largely top-down since the start of the conservation movement in Norway in the late 19^th^ century [47]. In the 1990s, local management of protected areas was first put on the political agenda by landowner organizations [16]. This was triggered by a national plan to create a number of new protected areas and expand existing ones, a plan that affected both productive and private land [14]. In 2009 the Norwegian Parliament approved a nationwide community-based conservation reform through a budgetary proposition [15]. The reform was a result of a successful mobilization of local politicians and a continuation of a general practice of delegating responsibilities from the central to the local level in Norway [48,49].

Before the reform different local governance arrangements were tested [14,50] with varied results [51–53]. The overall conclusion was that despite high local political commitment, local management responsibility did not manage to increase support for conservation in the local communities [50]. Evaluations also pointed out that there were few opportunities for real participation of stakeholders and other governmental agencies, and few arenas for conflict resolution [50].

### 1.2 The main actors in community-based conservation

**Fig 1:**
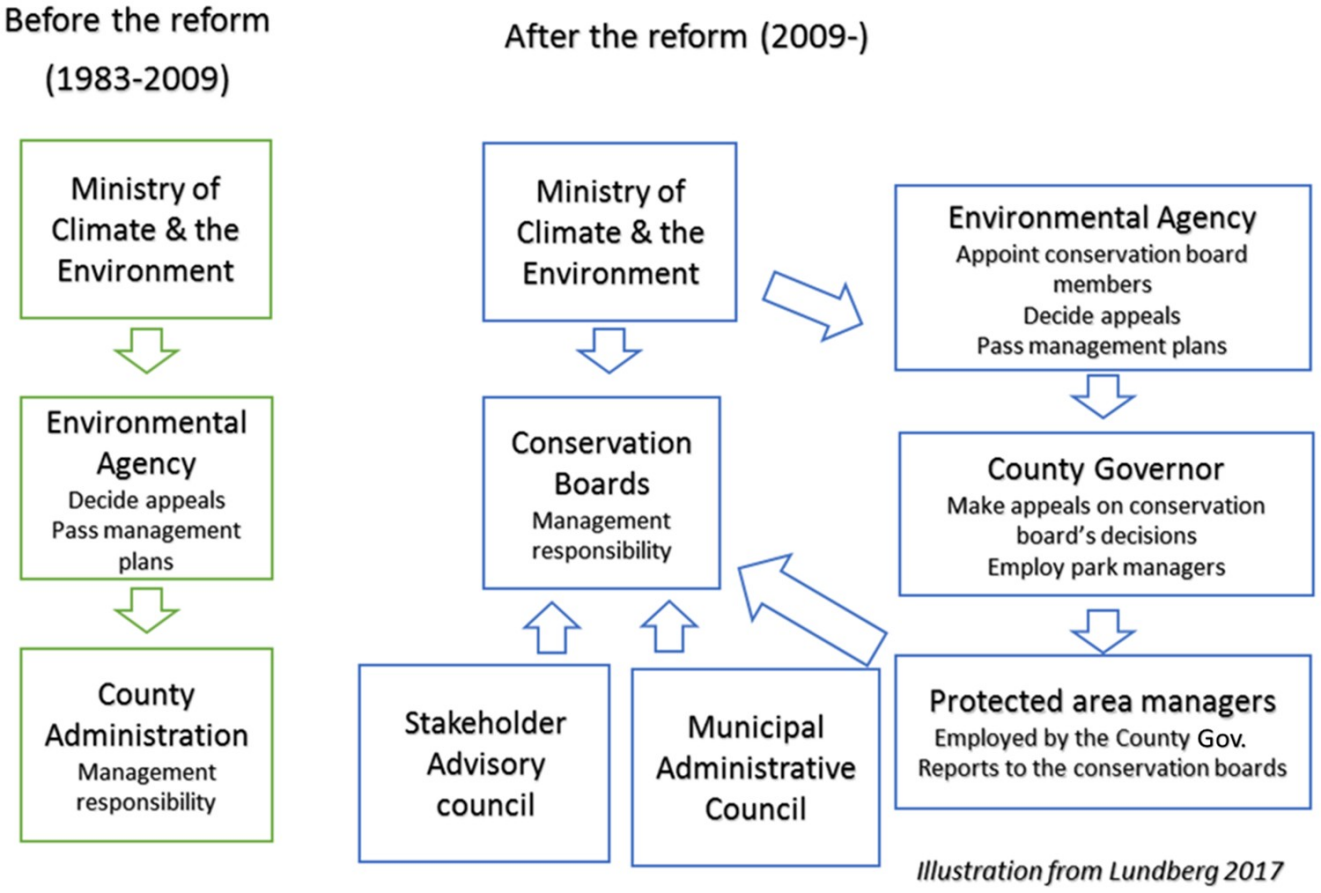
The main actors in Norwegian protected area governance before and after the reform. Local conservation boards are currently in charge of the daily decision-making. They appoint stakeholders to the advisory council that provides input to the local conservation board. The governmental bureaucracy at the regional level (i.e., the County Governor) held the management responsibility before the reform. The Environmental Agency is the professional agency at the national level, whereas the Ministry of Climate and Environment is the supreme political authority. Adapted from Lundberg [14] under a CC BY license, with permission from A. Lundberg original copyright 2017.

Local conservation boards were established as a result of the reform and these currently hold management responsibility for protected areas, a responsibility previously held by the government bureaucracy at the regional level (the County Governor; Fig 1). The members of these boards are for the most part local and regional politicians proposed by the municipalities and the County Parliament, and representatives proposed by the Sami Parliament. The conservation boards are officially appointed by the Environmental Agency–the professional bureaucracy at the national level. These boards currently manage single PAs or clusters of PAs, aided by park managers with conservation expertise employed as a result of the reform. The conservation boards are in charge of decisions regarding permit-applications, budgets, management plans, plans for management measures and other tasks [54]. The reform has resulted in the involvement of around 500 local and regional politicians in PA management and the employment of approximately 54 park managers. In total, 36 out of 39 Norwegian national parks as well as a substantial portion of protected landscapes and other protected areas are now governed locally.

The conservation boards’ decision-making is a result of interactions between multiple levels of governance. For instance, the boards must forward all their decisions to the County Governor and the Environmental Agency and report to the County Governor annually. The County Governor should ensure that conservation boards’ decisions follow the Nature Diversity Act and can appeal them on behalf of the government. Management plans and plans for management measures must be approved by the Environmental Agency. The Ministry of Climate and Environment is the supreme political authority for protected areas and can change the conservation boards’ mandate and retract the delegated authority if they do not comply with the overall goals of management [15]. The Environmental Agency and the Ministry can overturn conservation boards’ decisions if they consider them invalid (Public Administration Act § 35), but this has rarely been done in practice [55].

The conservation boards’ statutes give them room to decide on how to organize stakeholder participation through advisory councils and who to involve. The only requirement is that they appoint stakeholders that represent different interest groups in the protected area(s), and that they have at least one annual dialogue meeting with the stakeholder advisory council. Reports from these meetings are generally made publicly available online. Interest groups can include property owners and other rights holders (e.g., reindeer herders), commercial interests (e.g., tourism, hydropower industry), nature conservation—(e.g., Friends of the Earth, people employed at national park centers), recreation- (e.g., the National Trekking Association, Forum for Nature and Outdoor Recreation), hunting & fishing organizations (e.g., Norwegian Association of Hunters and Anglers) and village associations.

### 1.3 The status and conservation policy of Norwegian protected areas

The goals of Norwegian protected areas are to maintain natural variation of habitat types and landscapes, biodiversity, areas for small-scale outdoor recreation, natural and cultural history, ecological connectivity and reference areas (Nature Diversity Act § 33). Norway also has international commitments to conserve biodiversity through protected areas, for instance through the ratification of the UN Convention of Biological Diversity [56]. Each protected area has a set of rules (protection regulations) tailored to the local conditions during PA establishment. Non-motorized, low-impact access, small-scale harvesting and grazing are allowed in most protected areas [18,55,57]. Motorized vehicle use is mainly regulated through permits, whereas land development inside protected areas is generally either not allowed or also regulated through permits [58]. Property owners should be informed and involved during the process of protected area designation (Nature Diversity Act §§ 41–46) and they are financially compensated for the restrictions on use (Nature Diversity Act §51). They also retain hunting and fishing rights.

In the years leading up to the reform, reports from the County Governor showed the conservation values of an increasing proportion of protected areas were threatened by at one or more human activities [15,20]. The main threats to protected areas were alien species (mainly non-native species of wood), land abandonment, motorized vehicle use, other forms of traffic, land development and pollution [20]. The main reason for the inadequate management was a lack of manpower at the County Governor’s office [20].

Protected areas is one out of many measures used to safeguard biodiversity in Norway [59]. It is generally the policy instrument most strongly associated with nature conservation by the public and therefore the focus of this paper.

## 2. Research design and method

### 2.1 Study participants and study areas

**Fig 2:**
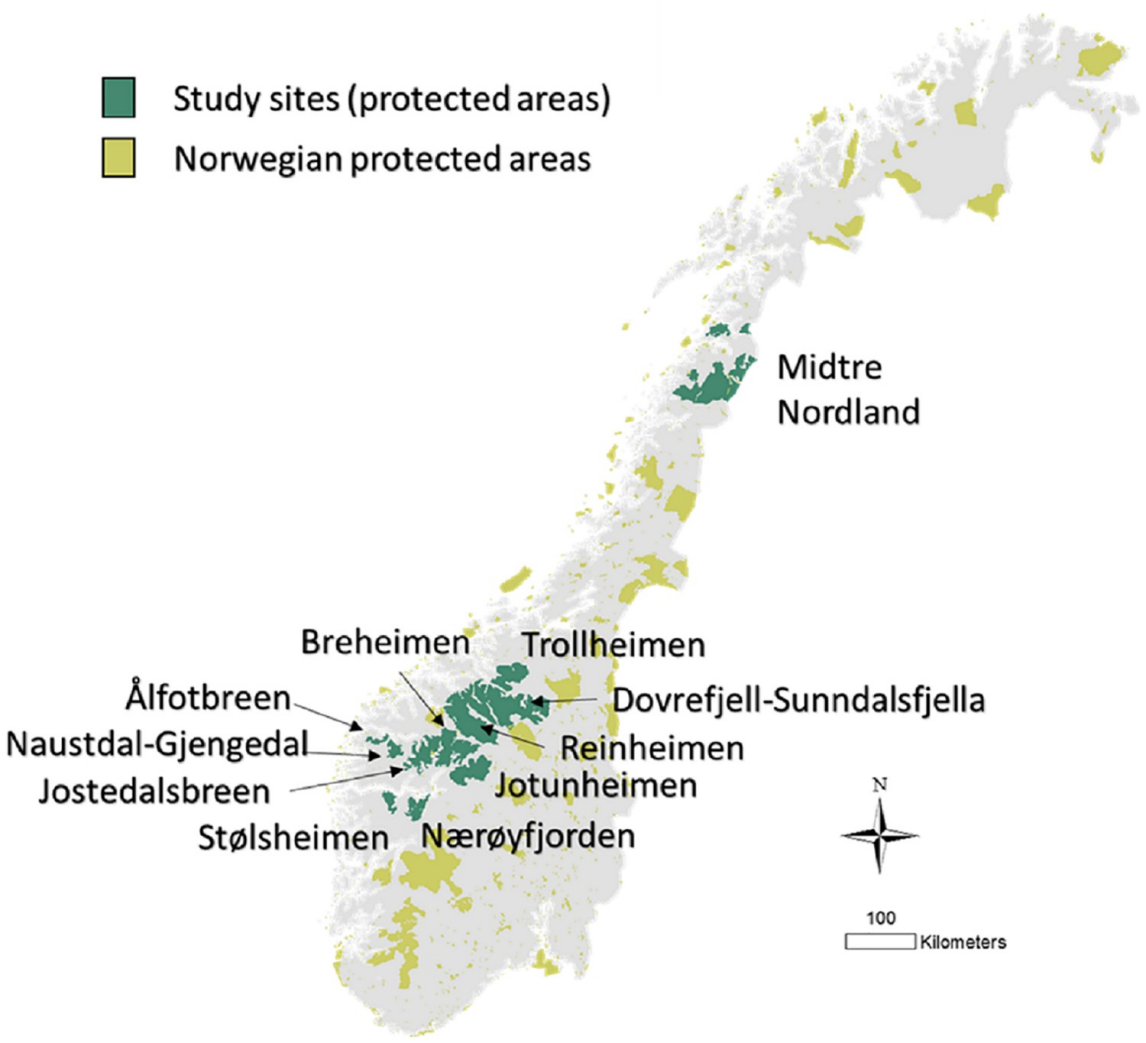
Study areas. Map showing the protected areas in Norway (light green/yellow) and the protected areas included in this study (dark green). Names refer to the different local conservation boards (and advisory councils as there is one advisory council for each conservation board) in charge of managing the PAs in the study areas. These either manage single protected areas like in Stølsheimen or clusters of protected areas like in Midtre-Nordland. The map was produced using ArcGIS version 10.5. The map sources were available online under a CC BY 4.0 license at [61,62].

The study participants were members of stakeholder advisory councils appointed by the conservation boards. The stakeholders represented on the advisory councils vary somewhat between areas, since the conservation boards are relatively free to decide. In some areas stakeholder advisory councils were present before the reform and continued under the new management authority. We invited all the members of 11 different stakeholder advisory councils to participate; one large advisory council covering 14 protected areas in Northern Norway and ten councils covering 42 protected areas in the south (Fig 2, S1 Table). Most of these study areas were selected as part of a larger research project CultES (https://arcticsustainability.com/2017/04/03/cultes/) to capture contrasts between urban and rural areas, private and public land and northern and southern Norway. These stakeholders were involved in managing 18353km^2^, which is ~33% of the total protected area on the Norwegian mainland (by November 2018, 55804 km^2^ of the Norwegian mainland was protected [60])

### 2.2 The survey

We developed a questionnaire to elicit stakeholders’ perceptions about the conservation problem operationalized as 1) perceived threats to conservation values, 2) management priorities (what should be prioritized/protected/allowed in PAs) and 3) who they would rely on to decide on conservation issues (trust in the different protected area governance actors), along with the interest group(s) they represent. The participants could rate nine categories of threats and their level of trust in the seven main actors involved in protected area governance on a five-point Likert scale from very low to very high, in addition to a no opinion option. They could further select between 16 management actions and check those that would be their main priorities if they held management responsibility (they could check all the management actions that applied to them, but we asked them to limit their answer to three). An open category where the participants could add other threats and priorities was also available. The threat categories that the participants were asked to evaluate were similar to the categories of activities reported to be negatively affecting conservation values in Norwegian protected areas prior to the initiation of a nationwide community-based conservation reform in 2009 [20]. These threats also correspond to standard threat categories known to affect conservation targets [63]. The priority questions were developed to elicit differences in perceptions among the participants about what should be protected along the nature-culture continuum and perceptions of the acceptable degree of human use (i.e., use vs protection) based on previous work on Norwegian protected areas [13,17,24,55].

As the community-based conservation reform aspired to increase support for protected areas we inquired about the participants’ attitudes towards PAs. We used a standard survey question developed and used by the European Commission to assess people’s attitudes towards conservation in their general population Eurobarometer survey [64]. The question focuses on the willingness to accept tradeoffs between conservation and economic development. The Eurobarometer survey results provides a benchmark for comparison in the absence of knowledge of stakeholders’ attitudes prior to the community-based conservation reform.

To assess preferences for overarching conservation ideas we asked the participants to select one of four conservation approaches developed from Mace [32] (i.e., “nature for itself”, “nature despite people”, “nature for people” and “people and nature”; see Introduction) that mostly agreed with their own view of how conservation should be approached, including a no opinion option. A survey approach to the preferred rationale for nature conservation has been applied by others [65]. A caveat with this approach is that it reduces quite broad topics in conservation into four survey items, whereas participants’ views may be more nuanced. A lack of fit between perceptions and survey items may also be relevant for other questions in this study, but to reduce this risk we have included open categories, as mentioned. We also feel that a lack of understanding should be captured by the no opinion option provided.

At the end of the survey we asked for demographics (gender, age, education) and that they report how they perceived the survey (i.e., easy & understandable, demanding & understandable, demanding & difficult to understand, and an open category where they could comment more extensively). The questionnaire contained in total 33 questions. The subset of questions used in this study are presented in Table 1 and can be found in full in the appendix (S2 Text) both in the original language (Norwegian) and in English.

**Table 1:** Variables from questionnaire responses used in the statistical analysis (see section 2.3). No opinion and missing answers have been imputed. The no opinion option in the questionnaire is therefore not included as a variable level in this table. See supplementary information (S2 Text) for full questionnaire.

Variable group	Type	Variables
Interest group	Categorical (yes, no)	Property owners, Hunting and fishing, Livestock, Tourism, Recreation, Industry/forestry, Public authority, Cultural heritage, Nature conservation
Threats to conservation objectives	Continuous (Very low Low Moderate High Very high)	Disturbance in buffer zone Woodland expansion Alien species Climate-change Overharvesting Pollution Land development Motorized vehicle use Traffic
Attitudes towards loss/degradation of protected areas	Ordinal:	
	Forbid (1)	This is not acceptable because these are our most important nature protection areas
	Partly acceptable (2)	This is only acceptable when it is in the public’s interest and if the damage is fully compensated for
	Acceptable (3)	This is acceptable because economic development is necessary
Conservation approach	Categorical:	
	Nature for itself	Human activity is kept outside protected areas and nature is allowed to develop without interference
	Nature despite people	Environmental condition and threats are monitored, and populations managed to avoid negative effects of human activity as much as possible.
	Nature for people	The great diversity of benefits provided by nature which humans depend on should be mapped and the costs to society if we lose these benefits should be measured
	People and nature	Nature should, to a greater extent, be viewed as shaped by human use and focus should be placed on the interrelationships between nature and culture
Management priorities	Categorical (yes, no)	Reduce land development (e.g., houses, roads, power lines) Prevent further land development Reduce traffic in sensitive areas Increase biodiversity by protecting wilderness Prevent further loss of biodiversity Facilitate traditional recreation Facilitate nature-based environmentally friendly tourism Protect cultural heritage and -landscapes Facilitate modern recreation (e.g., kiting, alpine, rafting) Maintain traditional grazing and hay making Facilitate modern, economically sustainable farming Facilitate commercial tourism Facilitate access for disabled people Improve conditions for reindeer herding Balance economic development and environmental protection Secure the interests of land owners and other stakeholders
Trust in protected area governance actors	Continuous (Very low to very high)	Municipality, Conservation board, Park managers, County Governor, Environmental Agency, Ministry of Climate and Environment, Stakeholder Advisory Council members

The protected area managers in the study areas gave feedback on the questionnaire and provided contact information to council members. The survey took place from mid-March to June 2016. All the 201 participants on the advisory councils received an e-mail with instructions and a link to access the survey online and had three weeks for completion. The survey initially included a mapping section. To reduce the effort required by the participants and to increase the response rate, we decided to send the survey a second time only with the questionnaire. Participants could request a paper copy, which we sent per mail with prepaid postage. After the second three-week deadline passed, we telephoned those who had not completed the questionnaire as a reminder and sent a text message to those that we did not reach by telephone.

An ethical review of our project was approved by the Norwegian Centre for Research Data which is the Data Protection Official for Research for all the Norwegian universities and research institutes (LINKAGE No. 39396; nsd.uib.no/). We complied with the ethical rules under the Personal Data Act. Participants had to sign a consent form to participate in the study. This form stated the purpose of the study, informed that participation was completely voluntary and that participants could withdraw from the study at any time. It also explained how the data would be stored and reported, and that participation was completely anonymous. We also provided contact details and encouraged participants to notify us or the Data Protection Official about any concerns (see S2 Text for the consent form (in Norwegian)).

### 2.3 Statistical analyses

First, we explored survey responses by interest group, as well as overall percentages where relevant. Second, we tested, using chi-squared tests and the Fisher’s Exact test (when cell counts were lower than 5), if perceptions (threats, priorities and trust), conservation approaches and participant demographics (age, gender and education) were related with attitudes to PA loss or degradation. Third, we assessed the interrelationship between stakeholders’ perceptions of protected area management with a Multiple Factor Analysis (MFA), which included four groups of variables; namely interest group (9 variables), management priorities (16 variables), perceived conservation threats (9 variables) and trust in management authorities (7 variables).

Multiple factor analysis (MFA) can be used with groups of variables, continuous or categorical that are collected on the same unit of analysis (e.g., individuals). The aim is to discover the main underlying structure in the data that is grouped into sets of variables [66]. In other words, by using MFA we could include multiple indicators/items for each of the four groups of variables (e.g., stakeholder representation, trust, perceived threats and priorities) to discover how they are interrelated. This allowed us to identify the major patterns among stakeholders with respect to who they represent, what they perceive as threats to conservation objectives, how they would prioritize management, and which protected area authority they trust the most to represent their interests.

MFA is based on Principle Component Analysis (PCA) when groups of variables are quantitative and Multiple Correspondence Analysis (MCA) when they are qualitative. To standardize the influence of each set of variables they are weighted by dividing their values by the square root of the first eigenvalue obtained in the PCA/MCA [67]. The results of an MFA are multiple independent factors that each explain a decreasing proportion of the total variation [68,69]. There are several ways of selecting the number of dimensions that adequately reflects the variability in the data. We adhered to the rule of thumb that suggests selecting the dimensions with eigenvalues larger than 1 [68], which means that the dimension accounts for as much or more variance as a single variable.

The variables included in the MFA are presented in Table 1. We made small adjustments to the survey responses for the purpose of the statistical analysis, which are described in the following. For the few participants who reported interest groups other than the ones specified, we were able to merge them with existing categories. The open category related to management actions did not reveal actions that were not captured by the specified items. Imputed values were inserted for missing observations in the threat, trust and attitude categories (either because the participant chose the “no opinion” category or they did not to respond to the question). We used the median response as the imputed value. For the threat category we imputed 51 out of 747 answers. For the trust category we imputed 25 out of 581 answers. For the attitude question we imputed 7 out of 83 answers. In an MFA, the distance from the origin reflects the contribution of the variable to the dimension, i.e. increasing distance increases the contribution. Using the median as the imputed value will accordingly have little effect on the end results. Recognizing that threat and trust are ordinal variables we treated them as continuous and not categorical in this study, which was also done by Young et al. [70], as this almost doubled the variation explained in the first two dimensions while providing approximately the same results (see supporting information for MFA output with trust and threat as categorical variables, S1 Fig and S5 Table).

To interpret the resulting factor maps it is important to take into account how well the variables are represented by the MFA dimensions (cos^2^) and how much they contribute to the construction of the dimensions. Variables with a high cos^2^ (where 1 is the maximum) signifies that the element is well projected on the axis and that the distances between these elements can be interpreted [71]. We highlighted or plotted variables when the sum of the cos^2^ of the first two dimensions were equal to or larger than 0.5. The contribution of variables in accounting for the variability of a dimension are expressed in percentage. If the contribution of the variables is uniform, the expected value (%) would be 1/(number of variables)*100 [67]. Variables with a contribution larger than this cutoff are considered important in contributing to the dimension [67]. For the factor map with continuous variables we plotted all variables and highlighted those that were well represented (cos^2^ > = 0.5) and had a high contribution (more than expected if the contribution of variables was uniform). For the factor maps with the categorical variables we plotted these variables and removed the others (those with a low contribution and/or cos^2^ < 0.5) to aid interpretation as these figures contained many variables. The variables with the highest contribution to the first dimensions are responsible for the main variation in the dataset [67].

To visualize how attitudes and conservation approaches were related to the axes, we included them as supplementary variables. Supplementary variables are projected on the axes but are not involved in the construction of the dimensions. We also tested the relationship between the resulting MFA dimensions and attitudes to PA loss or degradation and conservation approaches using ordinal and multinomial regression (see S1 Text).

The MFA was performed with the packages FactoMineR ver. 1.36 [72] and factoextra [73] and using the statistical software R [74].

## 3. Results

### 3.1 Response rate and participant characteristics

We received 93 questionnaires and achieved a response rate of 46%, which is similar to the response rate of another recent study using a web-based survey involving stakeholder advisory councils in Norway (42%; see paper 2 in Lundberg [14]). We removed ten participants whose answers we consider too incomplete for the statistical analysis. The remaining dataset comprised of 83 respondents. Seven participants chose the paper version. Over half of the participants had higher education (67%), the average age was 55 years and most participants were men (71%, S3 Table). The high portion of male participants reflected the highly gender-biased representation on the advisory councils (see S1 Table). The participants’ average length of advisory council membership was 3.5 years (max. 17 years; S3 Table). Half (51%) had prior experience from protected area management. The interests of property owners were represented by 42% of the participants, followed by hunting/fishing and recreation (both 26%), livestock grazing (22%), tourism and conservation (both 18%), public authority and cultural heritage (both 10%) and industrial development (8%). This distribution of interest groups among the respondents was relatively similar to the distribution of interest groups among the population of all the members of the advisory councils included in the study (S2 Table). We assume that non-response bias is low due to the similarity in gender distribution and interest groups between our sample of respondents and the population of advisory council members, however we cannot rule it out as our sample may be skewed with regards to other factors such as age and education. Most of the participants found the survey easy to understand (91%, n = 90).

### 3.2 Attitudes to the loss or degradation of protected areas

According to 31% of the participants, the loss or degradation of protected areas was acceptable because economic development takes precedence, whereas 42% found this acceptable only when it is in the public’s interest and if the damage is fully compensated for. PA loss or degradation was not acceptable to 25%.

**Fig 3:**
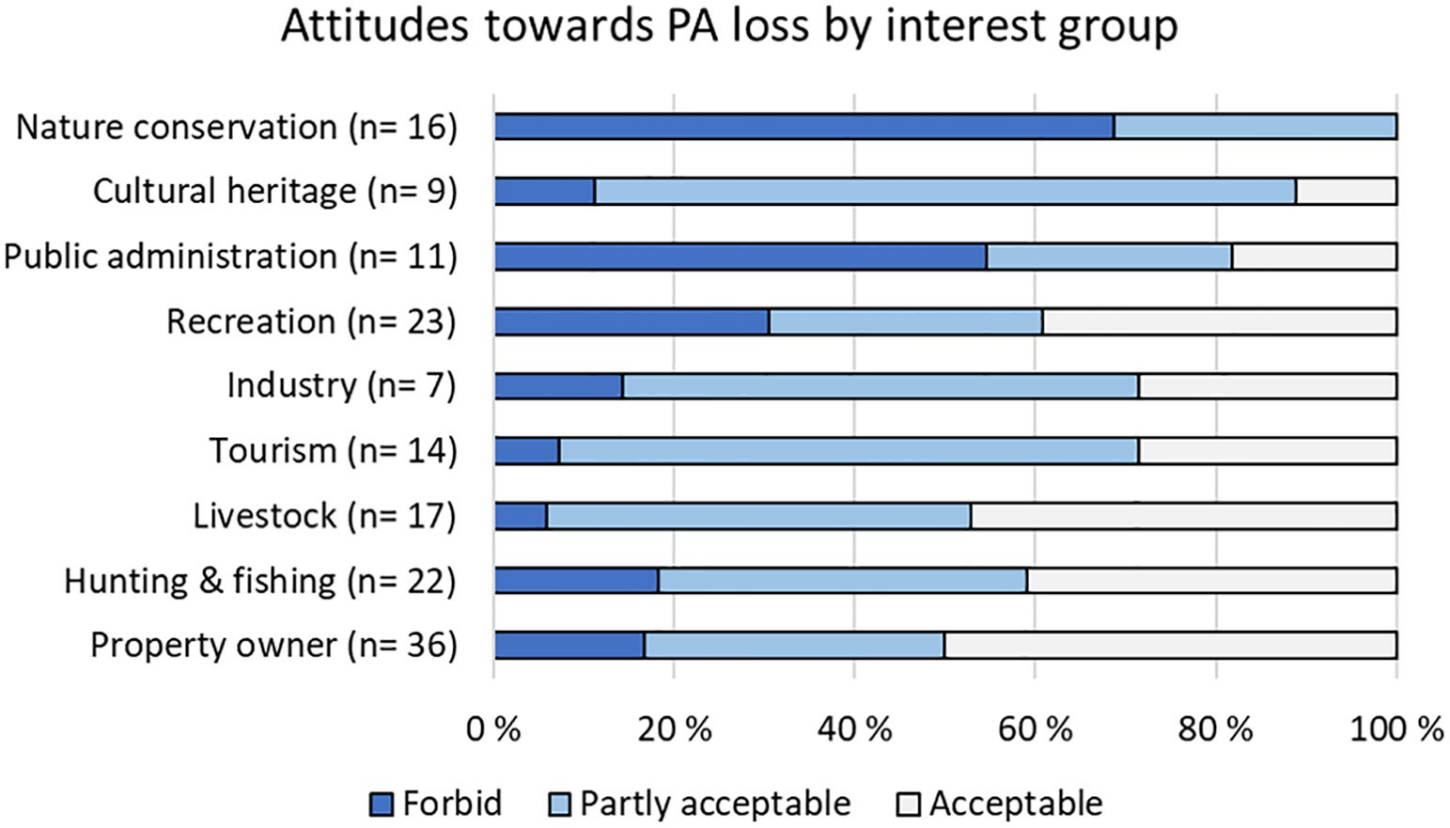
Support for protected areas. Attitudes towards the degradation or loss of protected areas for economic development by interest group, with the number of representatives within each interest group in parenthesis.

A large majority (>69%) within all interest groups except nature conservation and public administration found PA loss or degradation either partly acceptable or acceptable (Fig 3). PA loss or degradation was not acceptable to 69% of those representing nature conservation and 55% of those representing public administration (Fig 3). The interest groups where the highest proportion found PA loss or degradation acceptable was property owners (50%) followed by livestock owners (47%; Fig 3). There was no effect of gender, age or education (S9 Table).

### 3.3 Preferred conservation approach

Most participants chose “people and nature” (44%) or the “nature despite people” (47.6%) approach as a means of addressing environmental problems. “Nature for people” (6.1%) and “nature by itself” (2.4%) were selected by very few. Only one chose the no opinion option.

**Fig 4:**
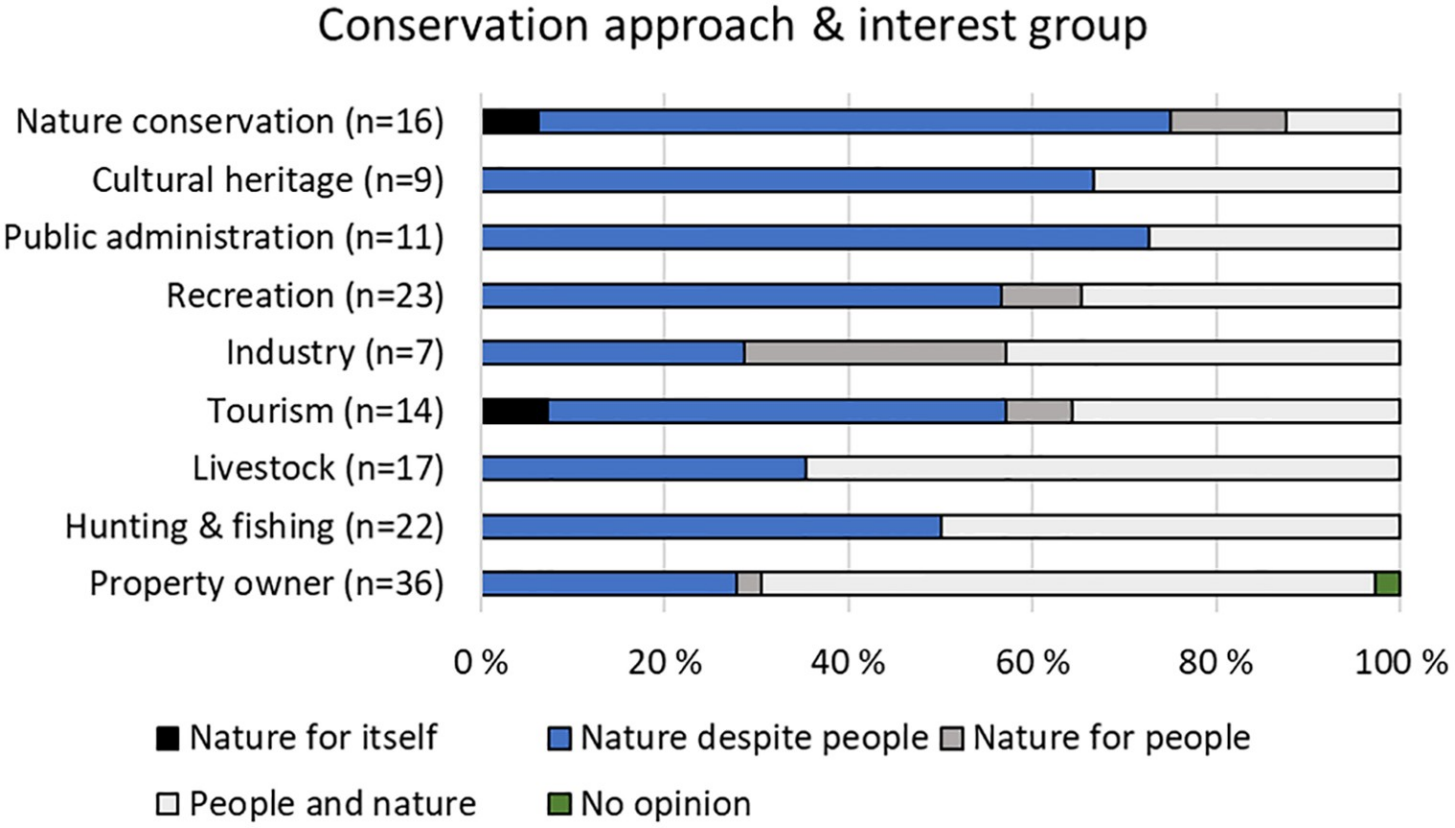
Preferred conservation approach. Perception of best way to approach conservation (i.e., through the “nature for itself”-, “nature despite people”-, “nature for people”- or the “people and nature” approach) by interest group, with the number of representatives within each interest group in parenthesis.

Many (>66%) of the participants representing nature conservation, cultural heritage and public administration favored the “nature despite people” approach, and >64% of the participants representing property owners and livestock favored the “people and nature” approach (Fig 4). The other interest groups were more divided (Fig 4)

### 3.4 Perceived threats to conservation values

Woodland expansion was perceived as a high to very high threat (59%), and overharvesting (86%), alien species (67%) and pollution (73%) was perceived as a very low or low threat by many participants. A high proportion (59%) of the participants found climate-change to be a threat to some degree.

**Fig 5:**
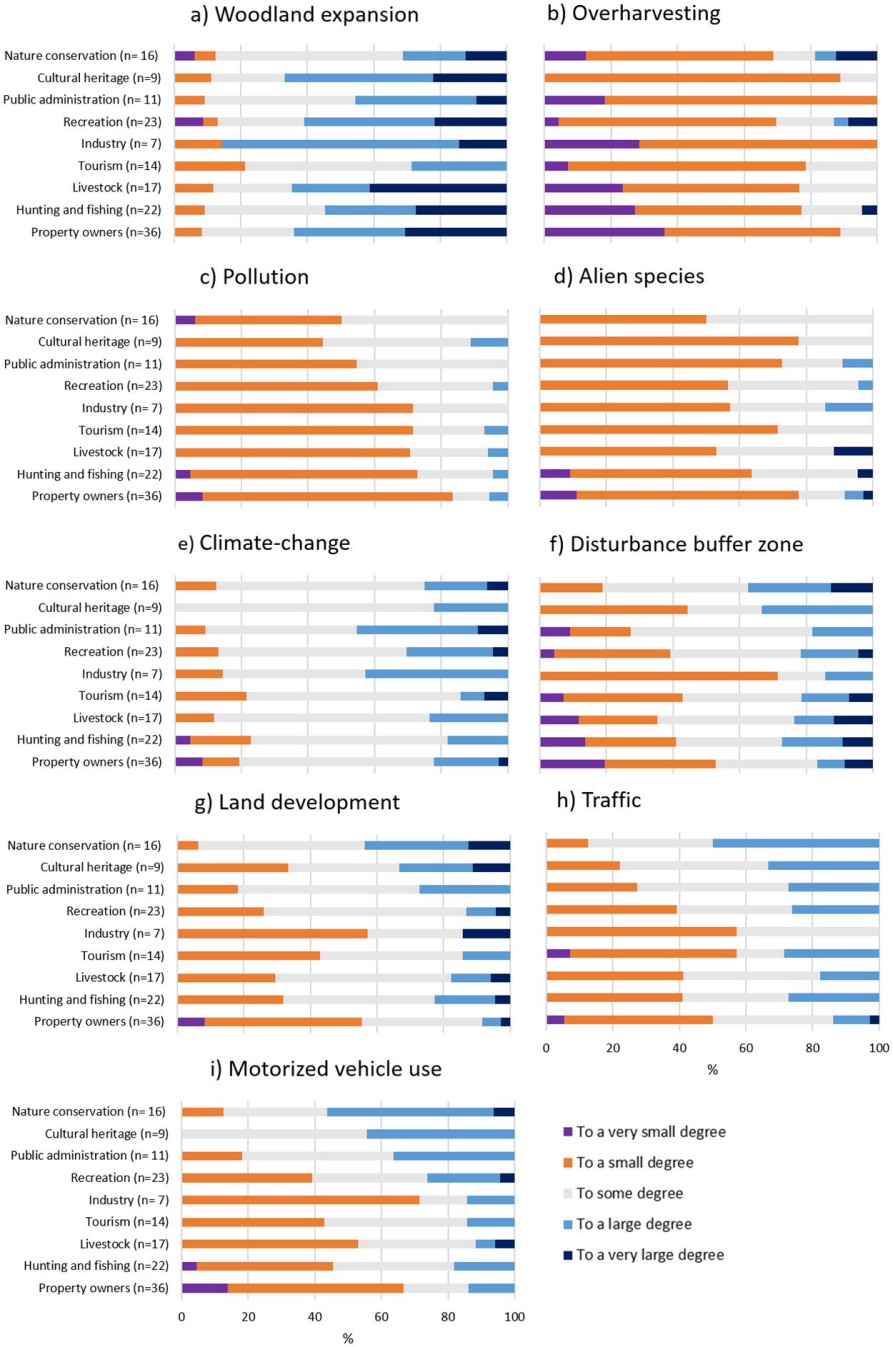
Perceived threats to conservation values. Participants’ threat assessments by interest group, with the number of representatives withing each interest group in parenthesis.

There was larger variation both between and within interest groups about whether land development (i.e., all types of human encroachments), motorized vehicle use, disturbance in the buffer zone and traffic vulnerable areas, were considered threats or not (Fig 5f–5i).

Perceived threats from land development, traffic and motorized vehicle use were also related with participants attitudes; those who found PA loss and degradation unacceptable rated these threats highest followed by those who found this partly acceptable and acceptable (in declining order; S6 Table).

### 3.5 Prioritized management actions

Maintaining grazing and hay making was prioritized by the highest proportion of the participants (48%), followed by traditional recreation (e.g., low impact activities like hiking, skiing, tenting with few technical encroachments; 36%), reducing traffic (e.g., motorized use, or on foot access/cycling/skiing) in vulnerable areas (34%), and maintaining biodiversity (33%).

Grazing and hay making was also a key priority for many interest groups. It was the most frequently selected priority of the interest groups livestock, cultural heritage, property owners, hunting and fishing, recreation and tourism (ranging from 50–71% of the participants within these interest groups). Halting land development (50%), reducing traffic (50%) and increasing biodiversity (50%) were the most frequently selected priorities among those representing nature conservation. Traditional recreation was selected by the highest number of the participants from public administration (55%) and maintaining biodiversity was selected by the most by participants representing industry (71%; see S12 Table for the distribution of management priorities by interest group).

Only the priorities modern farming and reduce land development were related with participants’ attitudes towards PA loss and degradation (S7 Table). Modern farming was prioritized by those who found PA loss and degradation acceptable and reducing land development by those who found it not acceptable.

### 3.6 Trust in protected area governance

**Fig 6:**
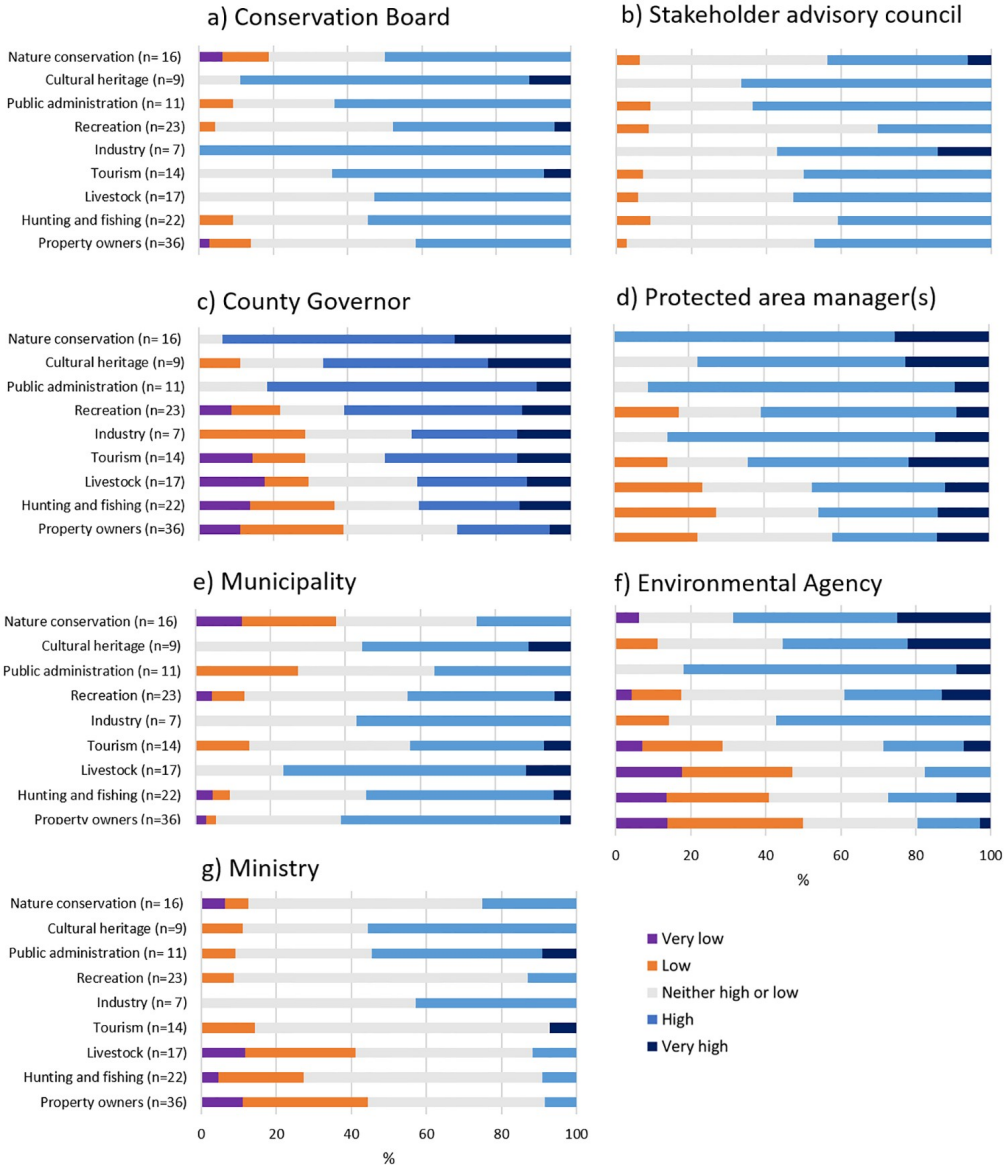
Trust in protected area governance. Participants’ level of trust in governance actors by interest group, with the number of representatives within each interest group in parenthesis.

The level of trust in the two governance actors established as a result of the reform, namely the stakeholder advisory councils and the conservation boards was relatively similar across and within interest groups, and most of the participants either rated their trust in these actors as high or as neither high or low (Fig 6a and 6b). Trust in the County Governor, the authority that held management responsibility before the reform, was more polarized, with participants reporting trust levels at both ends of the scale both within and between interest groups (Fig 6c). Trust in protected area managers (employed as a result of the reform) was overall high or very high, although a few participants from recreation, tourism, livestock, hunting and fishing, and property owners reported low trust in managers (Fig 6d).

Delegating management responsibility to the municipalities represents another alternative form of governance (along with the County Governor and the conservation boards). Trust in the municipality was not as polarized as in the County Governor. Nevertheless, trust in the municipality was lower than trust in conservation boards among several interest groups (nature conservation, cultural heritage, public administration, recreation, industry and tourism; Fig 6e). Trust in the environmental authorities at the national level, namely the Environmental Agency and the Ministry was relatively low among the participants from livestock, hunting and fishing and property owners compared with the other interest groups (Fig 6f and 6g).

Only trust in the County Governor was related with participants’ attitudes, where those who did not accept PA loss or degradation had higher levels of trust in the County Governor than those who found this acceptable (S8 Table).

### 3.7 Main differences in stakeholders’ perception—MFA results

**Fig 7:**
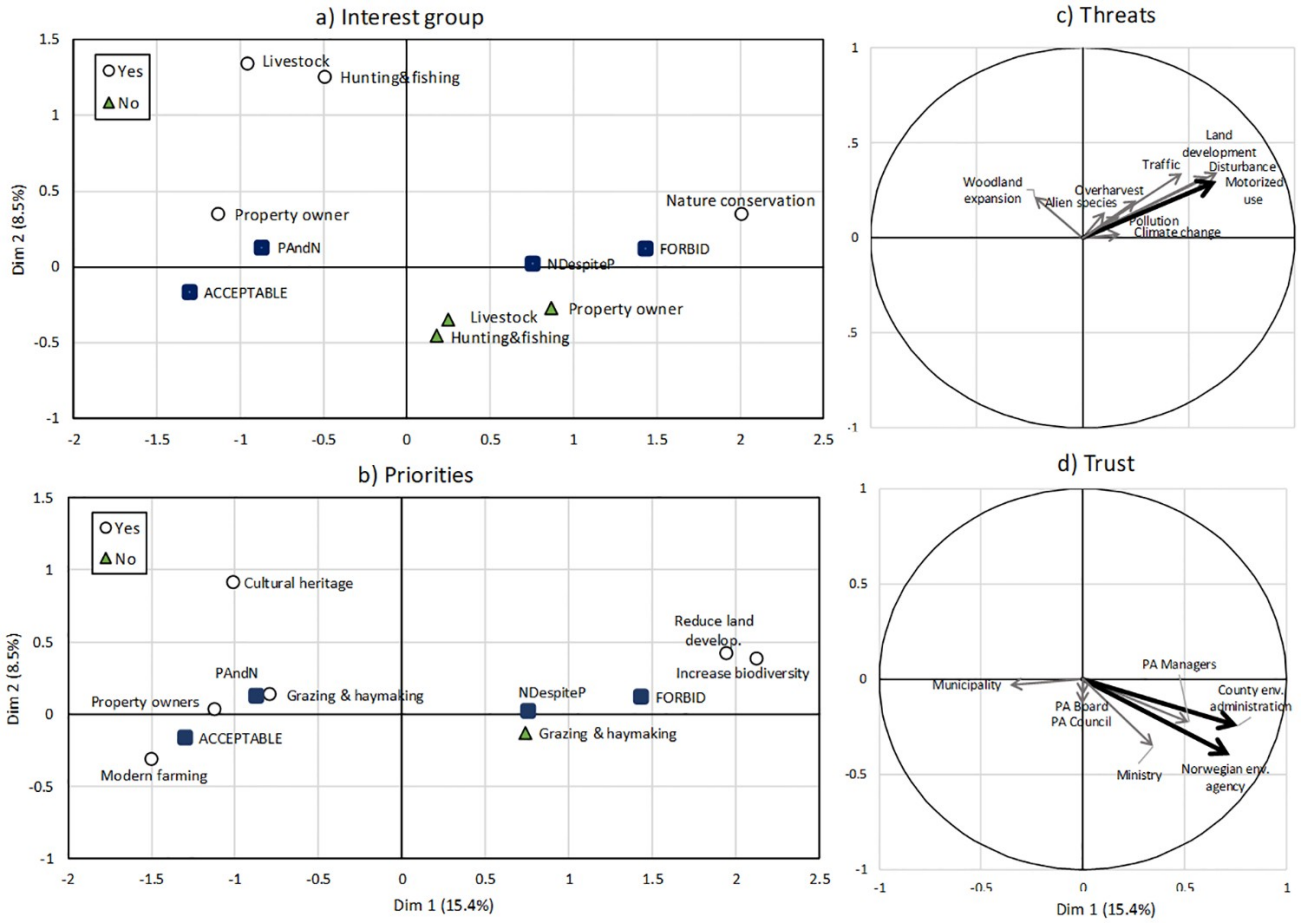
Divergent perceptions among stakeholders. Multiple factor analysis showing the two first dimensions and factor scores of the variables in the groups a) stakeholders, b) management priorities, c) conservation threats and d) trust in protected area governance actors. A and b show the output of the MFA for the categorical variables and c and d show the output for continuous variables. Factor scores for continuous variables are the correlations between the dimensions and the variables. In all plots, variables that are similar are grouped together and variables that are negatively related are positioned on opposite sides of the origin. Hollow circles in a and b represent participants who selected this variable and filled triangles those that did not. The supplementary variables conservation approach and attitudes are represented by filled squares. Only elements with a cos^2^ larger than or equal to 0.5 that contribute more than expected to the dimensions (see text for further explanation) were plotted in a and b. These variables are highlighted in c and d.

Only the first and second dimensions of the MFA analysis had eigenvalues above 1. These dimensions explained 15.4% and 8.5% of the total variation in the dataset comprising 4 groups and 66 variables. These two dimensions represented the most divergent perceptions among the local stakeholders. High values of dimension one reflected: i) nature conservation interests (Fig 7a); ii) management priorities associated with reducing land development and increasing biodiversity (Fig 7b), iii) perception of motorized vehicle use and disturbance in the buffer zone as major conservation threats (disturbance also has a relatively high contribution to the dimension and a cos^2^ of 0.47, i.e., just below the cutoff point 0.5; Fig 7c, S4 Table); and iv) high trust in regional (County Governor) and national environmental authorities (mainly the Environmental Agency; Fig 7d). Low values of dimension one reflected: i) property owners’ interests (Fig 7a); ii) the management priorities maintaining traditional grazing and hay making, securing the interests of property owners and other rights holders, and facilitating modern farming (Fig 7b); iii) perception of low levels of threats from motor use and disturbance (Fig 7c); and iv) lower trust in regional and national environmental authorities (Fig 7d). The interest groups hunting and fishing, and livestock grazing contributed to the second dimension (Fig 7a), along with the management priority cultural heritage (Fig 7b).

The two supplementary variables conservation approach (“nature for itself”, “nature despite people”, “nature for people”, and “people and nature”) and attitudes (forbid, partly acceptable and acceptable; filled squares in Fig 7a and 7b) were mainly associated with dimension 1, where a high value was associated with the “nature despite people” approach and an attitude to forbid PA loss or degradation, while a low value was associated with acceptance of PA loss or degradation and the “people and nature” approach. The associations between conservation approach and dimension 1, attitudes and dimension 1 and attitudes and approach were statistically significant. Gender and age were not significant explanatory variables. (see S1 Text, S10 and S11 Tables).

## 4. Discussion

### 4.1 Stakeholders’ perceptions & attitudes following the reform

The community-based conservation reform seems to have had relatively little effect on one of its main goals–increasing local support for protected areas, as local stakeholders’ attitudes towards PA loss or degradation were much more favorable than those found in general population surveys. For instance, the proportion who accepted the loss or degradation of protected areas was higher among our study participants than among European citizens surveyed in 2013 [64]. The survey included 25 573 respondents from various social and demographic groups in 28 European countries. Here PA loss or degradation was only acceptable to 9%, partly acceptable to 42% whereas 45% thought PA loss or degradation should be forbidden [64] (compared with this study where 31% found PA loss and degradation acceptable, 42% partly acceptable and 25% not acceptable). Similarly, Seippel et al. [75] found that 8.8% of Norwegians opposed biodiversity protection, 50% were neutral and 40.7% supportive (however the results are not directly comparable since the questions were posed differently in the two studies). We did not measure participants’ attitudes before the reform we cannot say if attitudes were less supportive than this and have improved as a result of the reform.

The perception of woodland expansion (i.e., the reforestation of cultural landscapes caused by climate change and altered land use practices) as a threat to conservation values found traction with many interest groups. This assessment was also reflected in their management priorities as many would prioritize facilitating livestock grazing and hay making. These perceptions align with the favorable attitudes towards livestock grazing among local people residing next to protected areas in Norway [76] and with Norwegians’ an affinity towards cultural landscapes [77]. They contrast with perceptions in other parts of the world where livestock grazing in PAs can be highly contentious due to livestock-wildlife interactions [78–81]. In Norway the loss of livestock to large carnivores (wolves, bears, lynx and wolverines) causes conflict, but this issue is decoupled from discussions about conservation policy in PAs as large carnivore conservation follows a different regulatory- and zonation scheme.

Stakeholders’ trust in the PA management authority that operated before the reform was highly polarized compared with the current conservation boards. Overall, trust in the three governance actors appointed as a result of the reform, namely conservation boards, stakeholder councils and protected area managers was relatively high. Having similar views of appropriate forums for management and dispute resolution is valuable for conflict management [82] and the results suggest that PA management may be in a better position to promote collaboration after the reform.

### 4.2 Lack of support & management implications

Conservation can mean different things to different people [33,83]. For instance, accepting PA loss and degradation for economic development does not necessarily mean that stakeholders do not support biodiversity conservation, where conservation can be broadly defined as “actions that are intended to establish, improve or maintain good relations with nature” [83]. Instead, it can imply that they prefer to preserve biodiversity through other means than protected areas. For many of the property owners in this study, PA management should focus on the threat of woodland expansion and facilitate human activity to combat this development. They were less concerned with activities such as land development and motorized vehicle use. Restricting some forms of human activity (e.g., land development, motorized vehicle use, pollution) is however, central to the current conservation policy in Norwegian PAs and the efforts made by the Norwegian government to improve support for PAs through community-based conservation without changing these PA regulations seems to have had little impact.

The lack of support for PAs among stakeholders could partly be due to the participatory process, which has been awarded relatively little attention compared with the reform’s ambitious goals of creating local ownership [15,84]. Conservation boards are, as mentioned (section 1.2), only required to have one meeting a year with the stakeholder advisory councils and some have not even met this requirement. The direct contact between stakeholders and conservation board members is modest and currently, conservation board members have a much more favorable evaluation of the functioning of stakeholder advisory councils than the stakeholders who serve on them [84]. It is well known that participatory processes done as a formality without the intention of informing decision making can be counterproductive [85].

Also at the heart of conservation conflicts is the fair distribution of costs and benefits between local people whose culture and livelihoods are tied to land use restricted by PAs and people elsewhere who benefit from the fulfillment of national and global conservation objectives. Adequate economic compensation can be a part of the solutions. For instance, economic compensation for voluntary forest conservation has been supported by forest owners in Norway in later years [86,87], who receive a relatively good price for their land and are free to accept or reject the offer made. Economic compensation may however be rejected by local people who find other values, such as heritage, more important. Economic compensation can also crowd out intrinsic, self-sustaining motivation for environmental behavior [88–90].

Other studies have found a lack of support for conservation to be associated with Norwegians’ views of nature as resilient rather than fragile, a lack of trust in science, favorable attitudes towards local-decision making, dependency on natural resources, gender, age and education (women, young and educated people more in favor) [75,91–95]. We confirm some of these results as large portion of property owners and livestock farmers (stakeholders that are likely to have higher resource dependency) were less supportive protection and had lower trust in higher-level environmental authorities. We did not find an effect of gender, age and education on attitudes in our study. This could be due to the relatively low sample size of our study compared with the studies cited herein that showed an effect.

If the goal is to reflect the attitudes of the general population and maintain a balanced representation of conservation and local interests on the stakeholder advisory councils, then attention to representation in PA management seems warranted. Other studies have shown how women and men use nature differently [96] and have different preferences for land management [76], suggesting that the current underrepresentation of women might have an impact on decision making. Political affiliation is also related with attitudes towards conservation [91,93]. Future studies should look at the perceptions and attitudes among the political representatives on the local conservation boards to assess potential implications of political representation vs stakeholder representation in decision-making.

Considering that a lack of resources was a key factor for poor conservation outcomes before the reform and that the initial trials of local management also showed little improvement in the local perception of conservation [50], the advice of the Environmental Agency to maintain responsibility at the regional level (County Governor), employ park managers stationed locally, establish advisory councils and increase financial and administrative resources [97] seems pertinent. On the other hand, institution building at the local level takes time [98]. Studies show that the local conservation boards (i.e., local and regional politicians) are good at considering local needs and adhering to conservation regulations at the same time [12]. Thus, focusing on the participatory process appears to be the way forward in order to create a greater understanding and support for the current PA policy.

Recently, attention has been devoted to relational values as a new way of motivating people to engage in conservation actions [99]. Recent research show that relational value statements that emphasize kinship with plants and animals, stewardship and responsibility to nature as well as to other humans resonate broadly across stakeholder groups [100]. As many stakeholders in this study seemed supportive of a relational approach, exploring the potential of relational values as a point of departure for engaging the stakeholder advisory councils in PA management in the future seems like a fruitful avenue.

## Supporting information

S1 Table Overview over the number of members and the gender balance on the advisory councils. (DOCX)

S2 Table The representation of interest groups on the advisory councils. (DOCX)

S3 Table Overview over participant demographics and membership characteristics. (DOCX)

S4 Table The quality of representation and contribution of the variables in the mixed MFA (where trust and threat variables were continuous). (DOCX)

S5 Table The quality of representation and contribution of the variables in the all-categorical MFA. (DOCX)

S6 Table Participants’ threat assessments by their attitudes towards protected areas. (DOCX)

S7 Table Participants’ management priorities by their attitudes towards protected areas. (DOCX)

S8 Table Participants’ trust in protected area governance actors by their attitudes towards protected areas. (DOCX)

S9 Table Participant demographics by their attitudes towards protected areas. (DOCX)

S10 Table Model selection of the regression models that assessed the relationship between conservation approaches, attitudes and MFA dimensions. (DOCX)

S11 Table Model output of the regression models that assessed the relationship between conservation approaches, attitudes and MFA dimensions. (DOCX)

S12 Table Management priorities by interest group. (DOCX)

S13 Table Survey data with imputed values. (XLS)

S1 Fig MFA results with trust and threat as categorical variables. (DOCX)

S1 Text Regression models of the associations between conservation approaches, attitudes and MFA dimensions—Methods and results. (DOCX)

S2 Text Survey questions (in English and Norwegian) and the survey consent form (in Norwegian only). (DOCX)
