# Supplementary material for: Stakeholders’ perceptions of protected area management following a nationwide community-based conservation reform
Source: PLoS One. 2019 Apr 24;14(4):e0215437. doi: 10.1371/journal.pone.0215437 (PMC6481814; doi:10.1371/journal.pone.0215437)
Supplement: S1 Table — (DOCX) [file pone.0215437.s001.docx]

Supporting information for: Stakeholders’ perceptions of protected area management following a nationwide community-based conservation reform

## Table S1. The number of members on each advisory council, the proportion of men and women or representation by an organization, in which case gender is not specified. Also shown is the number of survey respondents and the percentage of replies.

| Council | Members | % women | % men | % organization (no person specified) |  | Replies | % replies |
| --- | --- | --- | --- | --- | --- | --- | --- |
| Breheimen | 21^1^ | 48 | 48 | 5 |  | 8 | 38 |
| Dovrefjell | 28^2^ | 11 | 89 | 0 |  | 12 | 43 |
| Jostedalsbreen | 10 | 30 | 70 | 0 |  | 4 | 40 |
| Jotunheimen | 20 | 25 | 50 | 25 |  | 8 | 40 |
| Midtre-Nordland | 28^3^ | 18 | 82 | 0 |  | 15 | 54 |
| Naustdal-Gjengedal | 10 | 20 | 80 | 0 |  | 4 | 40 |
| Nærøyfjorden | 18^4^ | 33 | 56 | 11 |  | 4 | 22 |
| Reinheimen | 31 | 19 | 81 | 0 |  | 12 | 39 |
| Stølsheimen | 13 | 15 | 85 | 0 |  | 9 | 69 |
| Trollheimen | 14 | 21 | 79 | 0 |  | 7 | 50 |
| Aalfotbreen | 8^5^ | 13 | 75 | 13 |  | 3 | 38 |
| Other |  |  |  |  |  | 7 |  |
| Total | 201 | 23 (n=46) | 73 (n=146) | 4 (n=9) |  | 93 | 46 |

^1^Originally 22 members out of which 10 people are deputy board members. One informed that he was not a member anymore.

^2^Originally 30 members, but two informed that they were not involved anymore.

^3^Originally 29 members where 5 were deputy board members, but one informed that she was not involved anymore.

^4^Originally 20 members but two informed that they were not involved anymore.

^5^Originally 10 members but two informed that they were not involved anymore.
