# Supplementary material for: Stakeholders’ perceptions of protected area management following a nationwide community-based conservation reform
Source: PLoS One. 2019 Apr 24;14(4):e0215437. doi: 10.1371/journal.pone.0215437 (PMC6481814; doi:10.1371/journal.pone.0215437)
Supplement: S2 Table — (DOCX) [file pone.0215437.s002.docx]

Supporting information for: Stakeholders’ perceptions of protected area management following a nationwide community-based conservation reform

## Table S2. Representation by interest group on the advisory councils.

|  | Property owners | Hunting and fishing | Livestock grazing | Recreation | Tourism | Nature conservation | Cultural heritage | Public authority and education | Industry and forestry |
| --- | --- | --- | --- | --- | --- | --- | --- | --- | --- |
| Breheimen | 4 | 1 | 3 | 1 |  | 3 |  |  |  |
| Dovrefjell | 12 | 5 | 4 | 6 |  | 1 |  |  | 2 |
| Jostedalsbreen |  |  | 2 | 1 | 1 | 3 | 1 | 2 |  |
| Jotunheimen^1^ | NA | NA | NA | NA | NA | NA | NA | NA | NA |
| Midtre-Nordland | 3 |  | 6 | 2 | 2 | 1 |  | 10 |  |
| Naustdal-Gjengedal | 5 | 1 |  | 1 | 1 | 1 |  |  | 1 |
| Nærøyfjorden | 10 |  |  | 3 | 2 | 2 |  | 3 |  |
| Reinheimen | 14 |  | 2 | 5 | 2 |  | 3 |  | 5 |
| Stølsheimen | 5 | 1 |  | 1 |  | 1 |  | 4 | 1 |
| Trollheimen | 2 | 1 | 6 | 4 |  |  |  |  | 1 |
| Aalfotbreen | 3 |  |  | 1 | 2 | 1 |  | 1 | 2 |
| Sum | 58 | 9 | 23 | 25 | 10 | 13 | 4 | 20 | 12 |
| % of total | 33 | 5 | 13 | 14 | 6 | 7 | 2 | 11 | 7 |
| % representation by total number of study participants^2^ | 42 | 26 | 22 | 26 | 18 | 18 | 10 | 10 | 8 |
| % representation by total number of interest groups selected by participants^3^ | 23 | 14 | 12 | 14 | 9 | 9 | 5 | 7 | 5 |

^1^ The municipalities were in charge of appointing some of the representatives in Jotunheimen, and details about the interest groups of these members in are not known. ^2^These percentages do not add up to 100% because one participant could select more than one interest group. ^3^These percentages sum to app. 100% because it is the total number of times the interest group was selected divided by the total number of interests groups selected.
