# Supplementary material for: Stakeholders’ perceptions of protected area management following a nationwide community-based conservation reform
Source: PLoS One. 2019 Apr 24;14(4):e0215437. doi: 10.1371/journal.pone.0215437 (PMC6481814; doi:10.1371/journal.pone.0215437)
Supplement: S3 Table — (DOCX) [file pone.0215437.s003.docx]

Supporting information for: Stakeholders’ perceptions of protected area management following a nationwide community-based conservation reform

## Table S3. Demographics and membership characteristics of the study participants.

| **Gender** |  | **Education** |  | **Age (years)** |  | **Membership length (months)** |  | **Prior experience from PA management** |  |
| --- | --- | --- | --- | --- | --- | --- | --- | --- | --- |
| Female | 29 % | Higher | 67 % | 28-30 | 1 % | 0 | 1% | Yes | 51% |
| Male | 71 % | Secondary | 26 % | 31-40 | 8 % | 1-6 | 3% | No | 49% |
|  |  | Primary | 7 % | 41-50 | 19 % | 7-12 | 7% |  |  |
|  |  |  |  | 51-60 | 23 % | 13-24 | 15% |  |  |
|  |  |  |  | 61-70 | 26 % | 25-36 | 27% |  |  |
|  |  |  |  | 71-76 | 6 % | 37-48 | 24% |  |  |
|  |  |  |  |  |  | 49-100 | 16% |  |  |
|  |  |  |  |  |  | 101-204 | 5% |  |  |
|  |  |  |  |  |  |  |  |  |  |
|  |  |  |  |  |  |  |  |  |  |
| N = | 86 | N = | 86 | N = | 83 | N = | 91 | N = | 90 |
