# Supplementary material for: Stakeholders’ perceptions of protected area management following a nationwide community-based conservation reform
Source: PLoS One. 2019 Apr 24;14(4):e0215437. doi: 10.1371/journal.pone.0215437 (PMC6481814; doi:10.1371/journal.pone.0215437)
Supplement: S4 Table — (DOCX) [file pone.0215437.s004.docx]

Supporting information for: Stakeholders’ perceptions of protected area management following a nationwide community-based conservation reform

## Table S4. Representation and importance of variables in the MFA analysis in the main manuscript where trust and threat are defined as continuous variables. Variables included in the plot in the manuscript are highlighted in bold and defined as those with a sum of the cos^2^ of the two dimensions >= 0.5 (blue color) and a contribution for one or both of the dimensions larger than expected if the contribution of the variables was uniform (yellow color).

| Type of variable | Variable group | Variable | Sum cos2 | Dim 1 contribution | Dim 2 contribution |
| --- | --- | --- | --- | --- | --- |
| CATEGORICAL | STAKEHOLDER | **Nature.yes** | 0.815 | 6.635 | 0.666 |
|  | STAKEHOLDER | Nature.no | 0.815 | 1.584 | 0.159 |
|  | STAKEHOLDER | **Property.owner_Prop.yes** | 0.812 | 4.774 | 1.504 |
|  | STAKEHOLDER | **Property.owner_Prop.no** | 0.812 | 3.656 | 1.152 |
|  | STAKEHOLDER | **Lives.yes** | 0.734 | 1.599 | 10.314 |
|  | STAKEHOLDER | **Lives.no** | 0.734 | 0.412 | 2.657 |
|  | STAKEHOLDER | **Hunt.yes** | 0.706 | 0.554 | 11.574 |
|  | STAKEHOLDER | **Hunt.no** | 0.706 | 0.2 | 4.174 |
|  | PRIORITIES | **Renc.yes** | 0.699 | 4.435 | 0.478 |
|  | PRIORITIES | Renc.no | 0.699 | 0.678 | 0.073 |
|  | PRIORITIES | **Ibiod.yes** | 0.672 | 4.384 | 0.68 |
|  | PRIORITIES | Ibiod.no | 0.672 | 0.814 | 0.126 |
|  | PRIORITIES | **Mpast.yes** | 0.655 | 2.266 | 0.234 |
|  | PRIORITIES | **Mpast.no** | 0.655 | 2.108 | 0.218 |
|  | PRIORITIES | **Culth.yes** | 0.635 | 1.747 | 4.634 |
|  | PRIORITIES | Culth.no | 0.635 | 0.519 | 1.376 |
|  | PRIORITIES | **Property.owners_Prop.yes** | 0.565 | 2.603 | 0.008 |
|  | PRIORITIES | Property.owners_Prop.no | 0.565 | 0.998 | 0.003 |
|  | PRIORITIES | **Modfar.yes** | 0.541 | 2.619 | 0.356 |
|  | PRIORITIES | Modfar.no | 0.541 | 0.486 | 0.066 |
|  | STAKEHOLDER | Adm.yes | 0.423 | 0.923 | 4.609 |
|  | STAKEHOLDER | Adm.no | 0.423 | 0.141 | 0.704 |
|  | STAKEHOLDER | Recr.yes | 0.4 | 0.18 | 6.1 |
|  | STAKEHOLDER | Recr.no | 0.4 | 0.069 | 2.338 |
|  | PRIORITIES | Nrecr.yes | 0.36 | 0.481 | 4.229 |
|  | PRIORITIES | Nrecr.no | 0.36 | 0.024 | 0.214 |
|  | PRIORITIES | Rtraf.yes | 0.316 | 0.542 | 1.365 |
|  | PRIORITIES | Rtraf.no | 0.316 | 0.276 | 0.695 |
|  | PRIORITIES | Ctour.yes | 0.29 | 0.754 | 2.233 |
|  | PRIORITIES | Ctour.no | 0.29 | 0.019 | 0.055 |
|  | PRIORITIES | Senc.yes | 0.286 | 0.925 | 0.353 |
|  | PRIORITIES | Senc.no | 0.286 | 0.376 | 0.144 |
|  | PRIORITIES | Disab.yes | 0.272 | 0.867 | 0.899 |
|  | PRIORITIES | Disab.no | 0.272 | 0.068 | 0.07 |
|  | PRIORITIES | Trecr.yes | 0.179 | 0.335 | 0.746 |
|  | PRIORITIES | Trecr.no | 0.179 | 0.189 | 0.422 |
|  | PRIORITIES | Rein.yes | 0.165 | 0.714 | 0.143 |
|  | PRIORITIES | Rein.no | 0.165 | 0.036 | 0.007 |
|  | STAKEHOLDER | Cult.yes | 0.154 | 0.449 | 0.975 |
|  | STAKEHOLDER | Cult.no | 0.154 | 0.055 | 0.119 |
|  | STAKEHOLDER | Indu.yes | 0.149 | 0.003 | 2.628 |
|  | STAKEHOLDER | Indu.no | 0.149 | 0 | 0.242 |
|  | PRIORITIES | Balan.yes | 0.143 | 0.177 | 1.415 |
|  | PRIORITIES | Balan.no | 0.143 | 0.009 | 0.072 |
|  | PRIORITIES | Mbiod.yes | 0.109 | 0.214 | 0.551 |
|  | PRIORITIES | Mbiod.no | 0.109 | 0.103 | 0.266 |
|  | STAKEHOLDER | Tour.yes | 0.072 | 0.119 | 0.683 |
|  | STAKEHOLDER | Tour.no | 0.072 | 0.024 | 0.139 |
|  | PRIORITIES | Ntour.yes | 0.001 | 0 | 0.011 |
|  | PRIORITIES | Ntour.no | 0.001 | 0 | 0.004 |
|  |  |  |  |  |  |
| CONTINOUS | TRUST | **Env.agency** | 0.556 | 7.683 | 4.194 |
|  | THREAT | **Motorized.use** | 0.554 | 6.432 | 2.6 |
|  | TRUST | **County Governor** | 0.53 | 8.488 | 1.552 |
|  | THREAT | Land development | 0.493 | 5.512 | 3.084 |
|  | THREAT | Traffic | 0.472 | 3.528 | 3.415 |
|  | THREAT | Disturbance.border | 0.47 | 6.526 | 3.471 |
|  | TRUST | Managers | 0.39 | 3.995 | 1.33 |
|  | TRUST | Ministry | 0.326 | 1.747 | 3.31 |
|  | THREAT | Over.harvest | 0.177 | 1.032 | 1.158 |
|  | TRUST | Municipality | 0.175 | 1.884 | 0.027 |
|  | THREAT | Pollution | 0.132 | 0.497 | 0.363 |
|  | THREAT | Woodland expans. | 0.1 | 0.838 | 1.386 |
|  | THREAT | Alien.species | 0.051 | 0.182 | 0.562 |
|  | THREAT | Climate.change | 0.05 | 0.511 | 0.013 |
|  | TRUST | Council | 0.048 | 0 | 0.466 |
|  | TRUST | Board | 0.012 | 0 | 0.185 |
