# Supplementary material for: Stakeholders’ perceptions of protected area management following a nationwide community-based conservation reform
Source: PLoS One. 2019 Apr 24;14(4):e0215437. doi: 10.1371/journal.pone.0215437 (PMC6481814; doi:10.1371/journal.pone.0215437)
Supplement: S5 Table — (DOCX) [file pone.0215437.s005.docx]

Supporting information for: Stakeholders’ perceptions of protected area management following a nationwide community-based conservation reform

## Table S5. Representation and importance of variables in the MFA analysis with trust and threat as categorical variables. Variables included in the plot in S4 Fig are highlighted in bold and are defined as those with a sum of the cos^2^ of the two dimensions >= 0.5 (blue color) and a contribution for one or both of the dimensions larger than expected if the contribution of the variables was uniform (yellow color).

| Variable group | Variable | Sum cos2 | Dim 1 contribution | Dim 2 contribution |
| --- | --- | --- | --- | --- |
| STAKEHOLDER | **Property.owner_Prop.yes** | **0.797** | 5.02 | 1.223 |
| STAKEHOLDER | **Property.owner_Prop.no** | **0.797** | 3.845 | 0.937 |
| STAKEHOLDER | **Nature.yes** | **0.778** | 6.684 | 0.586 |
| STAKEHOLDER | **Nature.no** | **0.778** | 1.596 | 0.14 |
| STAKEHOLDER | **Hunt.yes** | **0.666** | 0.51 | 9.188 |
| STAKEHOLDER | **Hunt.no** | **0.666** | 0.184 | 3.314 |
| TRUST | **Env.agency_2** | **0.663** | 2.301 | 2.478 |
| STAKEHOLDER | **Lives.yes** | **0.646** | 1.52 | 7.767 |
| STAKEHOLDER | **Lives.no** | **0.646** | 0.392 | 2.001 |
| PRIORITIES | **Ibiod.yes** | **0.634** | 4.525 | 0.836 |
| PRIORITIES | **Ibiod.no** | **0.634** | 0.84 | 0.155 |
| THREATS | **Traffic_4** | **0.631** | 1.862 | 1.465 |
| TRUST | **County_2** | **0.619** | 3.6 | 0.123 |
| PRIORITIES | **Renc.yes** | **0.592** | 4.209 | 0.411 |
| PRIORITIES | Renc.no | 0.592 | 0.643 | 0.063 |
| PRIORITIES | **Mpast.yes** | **0.568** | 2.196 | 0.14 |
| PRIORITIES | **Mpast.no** | **0.568** | 2.043 | 0.131 |
| PRIORITIES | **Property.owners_Prop.yes** | **0.565** | 3.046 | 0.049 |
| PRIORITIES | **Property.owners_Prop.no** | **0.565** | 1.167 | 0.019 |
| THREATS | **Encroachment_2** | **0.549** | 1.312 | 0.499 |
| PRIORITIES | **Culth.yes** | **0.535** | 1.616 | 3.307 |
| PRIORITIES | **Culth.no** | **0.535** | 0.48 | 0.982 |
| THREATS | **Mortorized.use_4** | **0.527** | 1.713 | 0.248 |
| TRUST | **Managers_4** | **0.526** | 1.268 | 0.382 |
| TRUST | **Managers_2** | **0.521** | 2.416 | 0.813 |
| TRUST | **Env.agency_4** | **0.504** | 0.874 | 2.216 |
| THREATS | **Disturbance.border_4** | **0.5** | 1.335 | 0.397 |
| THREATS | Disturbance.border_1 | 0.496 | 1.976 | 0.104 |
| TRUST | Ministry_2 | 0.493 | 1.565 | 1.633 |
| PRIORITIES | Modfar.yes | 0.456 | 2.403 | 0.052 |
| PRIORITIES | Modfar.no | 0.456 | 0.446 | 0.01 |
| THREATS | Alien.species_3 | 0.451 | 0.536 | 1.234 |
| THREATS | Over.harvest_5 | 0.448 | 1.395 | 0.908 |
| THREATS | Mortorized.use_2 | 0.43 | 1.24 | 0 |
| STAKEHOLDER | Adm.yes | 0.425 | 1.007 | 4.254 |
| STAKEHOLDER | Adm.no | 0.425 | 0.154 | 0.65 |
| THREATS | Traffic_2 | 0.424 | 0.909 | 0.251 |
| STAKEHOLDER | Recr.yes | 0.421 | 0.346 | 5.551 |
| STAKEHOLDER | Recr.no | 0.421 | 0.133 | 2.128 |
| THREATS | Motorized.use_1 | 0.411 | 1.392 | 1.344 |
| TRUST | Env.agency_5 | 0.408 | 2.054 | 0.204 |
| TRUST | County_4 | 0.406 | 1.041 | 0.578 |
| TRUST | Ministry_4 | 0.359 | 0.536 | 2.335 |
| TRUST | Managers_3 | 0.348 | 1.12 | 0.374 |
| THREATS | Alien.species_1 | 0.344 | 1.188 | 0.151 |
| THREATS | Encroachment_4 | 0.317 | 0.937 | 0.222 |
| THREATS | Pollution_3 | 0.315 | 0.797 | 0.037 |
| THREATS | Climate.change_1 | 0.312 | 0.742 | 1.125 |
| PRIORITIES | Rtraf.yes | 0.306 | 0.599 | 1.347 |
| PRIORITIES | Rtraf.no | 0.306 | 0.305 | 0.686 |
| TRUST | Municipality_4 | 0.302 | 0.493 | 0.399 |
| TRUST | County_1 | 0.296 | 0.645 | 1.791 |
| THREATS | Disturbance.border_2 | 0.288 | 0.591 | 0.198 |
| THREATS | Overgrowth_5 | 0.287 | 0.224 | 1.124 |
| PRIORITIES | Ctour.yes | 0.282 | 0.771 | 2.121 |
| PRIORITIES | Ctour.no | 0.282 | 0.019 | 0.052 |
| TRUST | County_5 | 0.268 | 1.24 | 0.005 |
| PRIORITIES | Senc.yes | 0.263 | 0.896 | 0.517 |
| PRIORITIES | Senc.no | 0.263 | 0.365 | 0.21 |
| TRUST | Municipality_1 | 0.257 | 0.88 | 0.171 |
| PRIORITIES | Rein.yes | 0.249 | 1.311 | 0.532 |
| TRUST | Board_3 | 0.249 | 0.107 | 1.337 |
| PRIORITIES | Rein.no | 0.249 | 0.066 | 0.027 |
| PRIORITIES | Disab.yes | 0.248 | 0.765 | 1.673 |
| PRIORITIES | Disab.no | 0.248 | 0.06 | 0.13 |
| PRIORITIES | Nrecr.yes | 0.247 | 0.42 | 2.494 |
| PRIORITIES | Nrecr.no | 0.247 | 0.021 | 0.126 |
| TRUST | Council_4 | 0.241 | 0.013 | 1.369 |
| TRUST | Municipality_2 | 0.24 | 0.993 | 0.036 |
| TRUST | Env.agency_1 | 0.226 | 0.908 | 0.514 |
| THREATS | Over.harvest_4 | 0.226 | 0.451 | 0.166 |
| THREATS | Encroachment_1 | 0.221 | 0.58 | 0.508 |
| THREATS | Over.harvest_3 | 0.218 | 0 | 1.437 |
| THREATS | Climate.change_3 | 0.212 | 0.156 | 0.216 |
| THREATS | Pollution_4 | 0.211 | 0.007 | 1.313 |
| THREATS | Traffic_1 | 0.209 | 0.258 | 1.122 |
| THREATS | Overgrowth_3 | 0.208 | 0.418 | 0.005 |
| THREATS | Alien.species_4 | 0.202 | 0.015 | 1.129 |
| THREATS | Disturbance.border_3 | 0.197 | 0.356 | 0.031 |
| TRUST | Council_3 | 0.182 | 0.012 | 1.126 |
| PRIORITIES | Trecr.yes | 0.18 | 0.192 | 1.295 |
| PRIORITIES | Trecr.no | 0.18 | 0.109 | 0.733 |
| THREATS | Encroachment_5 | 0.174 | 0.715 | 0.16 |
| THREATS | Climate.change_2 | 0.165 | 0.366 | 0.014 |
| THREATS | Pollution_2 | 0.16 | 0.15 | 0.005 |
| THREATS | Over.harvest_2 | 0.157 | 0.001 | 0.459 |
| THREATS | Mortorized.use_3 | 0.142 | 0.332 | 0.001 |
| TRUST | Board_1 | 0.136 | 0.563 | 0.085 |
| THREATS | Overgrowth_1 | 0.131 | 0.337 | 0.011 |
| THREATS | Pollution_1 | 0.128 | 0.35 | 0.428 |
| TRUST | Managers_5 | 0.126 | 0.383 | 0.171 |
| TRUST | Board_4 | 0.122 | 0.033 | 0.534 |
| TRUST | Council_2 | 0.119 | 0.182 | 0.686 |
| THREATS | Encroachment_3 | 0.115 | 0.129 | 0.179 |
| STAKEHOLDER | Indu.yes | 0.111 | 0.001 | 1.773 |
| STAKEHOLDER | Indu.no | 0.111 | 0 | 0.163 |
| THREATS | Alien.species_5 | 0.102 | 0.209 | 0.369 |
| THREATS | Overgrowth_4 | 0.101 | 0.081 | 0.238 |
| THREATS | Disturbance.border_5 | 0.1 | 0.09 | 0.658 |
| TRUST | Ministry_3 | 0.099 | 0.203 | 0.014 |
| PRIORITIES | Mbiod.yes | 0.097 | 0.144 | 0.588 |
| PRIORITIES | Mbiod.no | 0.097 | 0.069 | 0.284 |
| TRUST | Ministry_1 | 0.093 | 0.4 | 0.114 |
| STAKEHOLDER | Cult.yes | 0.092 | 0.396 | 0.277 |
| STAKEHOLDER | Cult.no | 0.092 | 0.048 | 0.034 |
| THREATS | Over.harvest_1 | 0.09 | 0.246 | 0.01 |
| STAKEHOLDER | Tour.yes | 0.089 | 0.038 | 1.083 |
| STAKEHOLDER | Tour.no | 0.089 | 0.008 | 0.22 |
| TRUST | Municipality_3 | 0.072 | 0.011 | 0.369 |
| THREATS | Alien.species_2 | 0.063 | 0.001 | 0.138 |
| THREATS | Overgrowth_2 | 0.061 | 0.02 | 0.31 |
| TRUST | Council_5 | 0.058 | 0.222 | 0.024 |
| THREATS | Traffic_5 | 0.058 | 0.165 | 0.069 |
| THREATS | Climate.change_5 | 0.058 | 0.073 | 0.281 |
| TRUST | Municipality_5 | 0.051 | 0.172 | 0.135 |
| TRUST | Board_2 | 0.049 | 0.091 | 0.237 |
| THREATS | Motorized.use_5 | 0.048 | 0.1 | 0.138 |
| TRUST | Ministry_5 | 0.043 | 0.114 | 0.096 |
| PRIORITIES | Balan.yes | 0.038 | 0.052 | 0.339 |
| PRIORITIES | Balan.no | 0.038 | 0.003 | 0.017 |
| TRUST | County_3 | 0.035 | 0.096 | 0.001 |
| TRUST | Board_5 | 0.029 | 0.029 | 0.18 |
| TRUST | Env.agency_3 | 0.016 | 0.008 | 0.095 |
| THREATS | Traffic_3 | 0.006 | 0.005 | 0.006 |
| THREATS | Climate.change_4 | 0.006 | 0.001 | 0.031 |
| PRIORITIES | Ntour.yes | 0.003 | 0.005 | 0.017 |
| PRIORITIES | Ntour.no | 0.003 | 0.002 | 0.007 |
