# Supplementary material for: Stakeholders’ perceptions of protected area management following a nationwide community-based conservation reform
Source: PLoS One. 2019 Apr 24;14(4):e0215437. doi: 10.1371/journal.pone.0215437 (PMC6481814; doi:10.1371/journal.pone.0215437)
Supplement: S6 Table — (DOCX) [file pone.0215437.s006.docx]

Supporting information for: Stakeholders’ perceptions of protected area management following a nationwide community-based conservation reform

## Table S6. Participants’ threat assessments separated by attitudes towards PA loss or degradation (forbid = 22, partly acceptable n = 35, acceptable n = 26). Numbers are percentages. Statistically significant differences are estimated using Fisher’s exact test. Significance: *** P< 0.001, ** P < 0.01, * P < 0.05, . P < 0.1. The question was “to what degree do you believe that the conservation values are threatened by the categories listed?”. They yellow rows represent significance at the 0.05 level following a Bonferroni correction for multiple comparisons.

|  | | **To a very small degree** | **To a small degree** | **To some degree** | **To a large degree** | **To a very large degree** | **Significance** |
| --- | --- | --- | --- | --- | --- | --- | --- |
|  | |  |  |  |  |  |  |
| Disturbance buffer zone | Forbid | 0.0 | 18.2 | 36.4 | 31.8 | 13.6 | 0.0030** |
|  | Partly acceptable | 5.7 | 34.3 | 37.1 | 14.3 | 8.6 |  |
|  | Acceptable | 26.9 | 34.6 | 38.5 | 0.0 | 0.0 |  |
|  |  |  |  |  |  |  |  |
| Woodland expansion | Forbid | 4.5 | 9.1 | 45.5 | 27.3 | 13.6 | 0.6198 |
|  | Partly acceptable | 2.9 | 8.6 | 20.0 | 42.9 | 25.7 |  |
|  | Acceptable | 0.0 | 11.5 | 26.9 | 38.5 | 23.1 |  |
|  |  |  |  |  |  |  |  |
| Alien species | Forbid | 0.0 | 68.2 | 31.8 | 0.0 | 0.0 | 0.3799 |
|  | Partly acceptable | 2.9 | 62.9 | 22.9 | 5.7 | 5.7 |  |
|  | Acceptable | 11.5 | 57.7 | 19.2 | 11.5 | 0.0 |  |
|  |  |  |  |  |  |  |  |
| Climate change | Forbid | 4.5 | 4.5 | 54.5 | 22.7 | 13.6 | 0.1004 |
|  | Partly acceptable | 0.0 | 8.6 | 68.6 | 22.9 | 0.0 |  |
|  | Acceptable | 7.7 | 23.1 | 50.0 | 15.4 | 3.8 |  |
|  |  |  |  |  |  |  |  |
| Overharvesting | Forbid | 22.7 | 50.0 | 13.6 | 4.5 | 9.1 | 0.2243 |
|  | Partly acceptable | 17.1 | 68.6 | 14.3 | 0.0 | 0.0 |  |
|  | Acceptable | 26.9 | 69.2 | 3.8 | 0.0 | 0.0 |  |
|  |  |  |  |  |  |  |  |
| Pollution | Forbid | 4.5 | 54.5 | 36.4 | 4.5 | 0.0 | 0.1490 |
|  | Partly acceptable | 0.0 | 74.3 | 22.9 | 2.9 | 0.0 |  |
|  | Acceptable | 11.5 | 73.1 | 15.4 | 0.0 | 0.0 |  |
|  |  |  |  |  |  |  |  |
| Land development | Forbid | 0.0 | 4.5 | 50.0 | 31.8 | 13.6 | 0.0000*** |
|  | Partly acceptable | 0.0 | 40.0 | 37.1 | 14.3 | 8.6 |  |
|  | Acceptable | 11.5 | 53.8 | 34.6 | 0.0 | 0.0 |  |
|  |  |  |  |  |  |  |  |
| Motorized vehicle use | Forbid | 0.0 | 9.1 | 36.4 | 50.0 | 4.5 | 0.0000*** |
|  | Partly acceptable | 0.0 | 42.9 | 34.3 | 20.0 | 2.9 |  |
|  | Acceptable | 19.2 | 57.7 | 23.1 | 0.0 | 0.0 |  |
|  |  |  |  |  |  |  |  |
| Traffic | Forbid | 0.0 | 13.6 | 45.5 | 36.4 | 4.5 | 0.0016** |
|  | Partly acceptable | 2.9 | 42.9 | 31.4 | 22.9 | 0.0 |  |
|  | Acceptable | 7.7 | 53.8 | 38.5 | 0.0 | 0.0 |  |
