# Supplementary material for: Stakeholders’ perceptions of protected area management following a nationwide community-based conservation reform
Source: PLoS One. 2019 Apr 24;14(4):e0215437. doi: 10.1371/journal.pone.0215437 (PMC6481814; doi:10.1371/journal.pone.0215437)
Supplement: S7 Table — (DOCX) [file pone.0215437.s007.docx]

Supporting information for: Stakeholders’ perceptions of protected area management following a nationwide community-based conservation reform

## Table S7. Participants’ priorities separated by attitudes towards PA loss or degradation (forbid n = 22, partly acceptable n = 35, acceptable n = 26). Numbers are counts. Statistically significant differences are estimated using Fisher’s exact test in cell counts <5 and chi squared if not. Significance: *** P< 0.001, ** P < 0.01, * P < 0.05, . P < 0.1. Only priorities selected by more than 10 participants are included to focus on those of greatest importance and reduce the number of statistical tests. They yellow rows represent significance at the 0.05 level following a Bonferroni correction for multiple comparisons.

|  |  | **Forbid** | **Partly acceptable** | **Acceptable** | **Test** | **P-value** |
| --- | --- | --- | --- | --- | --- | --- |
| Modern farming (n=13) | Yes | 0 | 1 | 12 | Fisher's | 0.0000*** |
|  | No | 22 | 34 | 14 |  |  |
| Cultural heritage (n=19) | Yes | 2 | 10 | 7 | Fisher's | 0.1811 |
|  | No | 20 | 25 | 19 |  |  |
| Property owners (n= 23) | Yes | 3 | 8 | 12 | Fisher's | 0.0357* |
|  | No | 19 | 27 | 14 |  |  |
| Nature-based  tourism (n= 23) | Yes | 4 | 11 | 8 | Fisher's | 0.5252 |
|  | No | 18 | 24 | 18 |  |  |
| Maintaining grazing (n= 40) | Yes | 7 | 17 | 16 | Chi sq. | 0.12131 |
|  | No | 15 | 18 | 10 |  |  |
| Traditional  recreation (n= 30) | Yes | 6 | 13 | 11 | Chi sq. | 0.6926 |
|  | No | 16 | 22 | 15 |  |  |
| Maintain biodiversity (n= 27) | Yes | 7 | 11 | 9 | Chi sq. | 0.9627 |
|  | No | 15 | 24 | 17 |  |  |
| Reduce traffic (n= 28) | Yes | 10 | 13 | 5 | Chi sq. | 0.1367 |
|  | No | 12 | 22 | 21 |  |  |
| Stabilize land  development (n= 24) | Yes | 11 | 8 | 5 | Chi sq. | 0.0375* |
|  | No | 11 | 27 | 21 |  |  |
| Increase biodiversity (n= 13) | Yes | 7 | 6 | 0 | Fisher's | 0.0038** |
|  | No | 15 | 29 | 26 |  |  |
| Reduce land development (n= 11) | Yes | 8 | 3 | 0 | Fisher's | 0.0005*** |
|  | No | 14 | 32 | 26 |  |  |
