# Supplementary material for: Stakeholders’ perceptions of protected area management following a nationwide community-based conservation reform
Source: PLoS One. 2019 Apr 24;14(4):e0215437. doi: 10.1371/journal.pone.0215437 (PMC6481814; doi:10.1371/journal.pone.0215437)
Supplement: S8 Table — (DOCX) [file pone.0215437.s008.docx]

Supporting information for: Stakeholders’ perceptions of protected area management following a nationwide community-based conservation reform

## Table S8. Participants’ trust in governance actors separated by attitudes towards PA loss or degradation (forbid = 22, partly acceptable n = 35, acceptable n = 26). Numbers are percentages. Statistically significant differences are estimated using Fisher’s exact test. Significance: *** P< 0.001, ** P < 0.01, * P < 0.05, . P < 0.1. The question was “what is your level of trust in these protected area governance actors?”. The yellow rows represent significance at the 0.05 level following a Bonferroni correction for multiple comparisons.

|  |  | **Very low** | **Low** | **Neither high or low** | **High** | **Very high** | **Significance** |
| --- | --- | --- | --- | --- | --- | --- | --- |
| Municipality | Forbid | 13.6 | 27.3 | 31.8 | 27.3 | 0.0 | 0.012* |
|  | Partly acceptable | 0.0 | 5.7 | 34.3 | 54.3 | 5.7 |  |
|  | Acceptable | 0.0 | 3.8 | 34.6 | 57.7 | 3.8 |  |
|  |  |  |  |  |  |  |  |
| Conservation board | Forbid | 4.5 | 9.1 | 36.4 | 45.5 | 4.5 | 0.1361 |
|  | Partly acceptable | 2.9 | 2.9 | 22.9 | 68.6 | 2.9 |  |
|  | Acceptable | 0.0 | 11.5 | 50.0 | 38.5 | 0.0 |  |
|  |  |  |  |  |  |  |  |
| Managers | Forbid | 0.0 | 0.0 | 9.1 | 68.2 | 22.7 | 0.0222* |
|  | Partly acceptable | 0.0 | 11.4 | 20.0 | 45.7 | 22.9 |  |
|  | Acceptable | 0.0 | 26.9 | 30.8 | 34.6 | 7.7 |  |
|  |  |  |  |  |  |  |  |
| County Governor | Forbid | 0.0 | 4.5 | 9.1 | 63.6 | 22.7 | 0.0014** |
|  | Partly acceptable | 8.6 | 8.6 | 28.6 | 31.4 | 22.9 |  |
|  | Acceptable | 7.7 | 30.8 | 34.6 | 26.9 | 0.0 |  |
|  |  |  |  |  |  |  |  |
| Environmental Agency | Forbid | 4.5 | 13.6 | 18.2 | 50.0 | 13.6 | 0.0402* |
|  | Partly acceptable | 8.6 | 20.0 | 25.7 | 31.4 | 14.3 |  |
|  | Acceptable | 15.4 | 15.4 | 53.8 | 15.4 | 0.0 |  |
|  |  |  |  |  |  |  |  |
| The Ministry | Forbid | 4.5 | 18.2 | 54.5 | 22.7 | 0.0 | 0.087. |
|  | Partly acceptable | 11.4 | 14.3 | 48.6 | 20.0 | 5.7 |  |
|  | Acceptable | 3.8 | 23.1 | 57.7 | 15.4 | 0.0 |  |
|  |  |  |  |  |  |  |  |
| Advisory council | Forbid | 0.0 | 4.5 | 54.5 | 36.4 | 4.5 | 0.3564 |
|  | Partly acceptable | 0.0 | 2.9 | 31.4 | 62.9 | 2.9 |  |
|  | Acceptable | 0.0 | 3.8 | 50.0 | 46.2 | 0.0 |  |
