# Supplementary material for: Stakeholders’ perceptions of protected area management following a nationwide community-based conservation reform
Source: PLoS One. 2019 Apr 24;14(4):e0215437. doi: 10.1371/journal.pone.0215437 (PMC6481814; doi:10.1371/journal.pone.0215437)
Supplement: S9 Table — (DOCX) [file pone.0215437.s009.docx]

Supporting information for: Stakeholders’ perceptions of protected area management following a nationwide community-based conservation reform

## Table S9. Participants’ demographics by attitudes towards PA loss or degradation. Numbers are counts. Statistically significant differences are estimated using ^1^Fisher’s exact test and ^2^Chi. Square test. Significance: *** P< 0.001, ** P < 0.01, * P < 0.05, . P < 0.1.

|  |  | **Forbid** | **Partly**  **acceptable** | **Acceptable** | **P-value** |
| --- | --- | --- | --- | --- | --- |
| Gender | Female | 6 | 9 | 9 | 0.7355^2^ |
|  | Male | 16 | 26 | 17 |  |
|  |  |  |  |  |  |
|  |  |  |  |  |  |
| Age | 28 to 40 | 2 | 3 | 4 | 0.713^1^ |
|  | 41 to 50 | 6 | 7 | 7 |  |
|  | 51 to 60 | 6 | 9 | 9 |  |
|  | 61 to 76 | 8 | 16 | 6 |  |
|  |  |  |  |  |  |
| Education | Higher | 15 | 16 | 22 | 0.3123^2^ |
|  | Primary&  secondary | 11 | 6 | 7 |  |
|  |  |  |  |  |  |
