# Supplementary material for: Stakeholders’ perceptions of protected area management following a nationwide community-based conservation reform
Source: PLoS One. 2019 Apr 24;14(4):e0215437. doi: 10.1371/journal.pone.0215437 (PMC6481814; doi:10.1371/journal.pone.0215437)
Supplement: S10 Table — (DOCX) [file pone.0215437.s010.docx]

Supporting information for: Stakeholders’ perceptions of protected area management following a nationwide community-based conservation reform

## Table S10. Model selection using backwards elimination.

|  | **Model** | **AICc** | **Removed variable** |
| --- | --- | --- | --- |
| Attitudes and MFA | ATTITUDES~ AGE + GENDER + MFA.DIM1 + MFA.DIM2 | 140.59 | Full model |
|  | ATTIUTDES ~ GENDER + MFA.DIM1 + MFA.DIM2 | 138.26 | AGE |
|  | ATTIUTDES ~ MFA.DIM1 + MFA.DIM2 | 136.25 | GENDER |
| Attitudes and conservation approach | ATTIUTDES ~ AGE + GENDER + APPROACH | 171.69 | Full model |
|  | ATTIUTDES ~ AGE + APPROACH | 169.49 | GENDER |
|  | ATTIUTDES ~ APPROACH | 167.97 | AGE |
| Conservation approach and MFA | APPROACH ~ AGE + GENDER + MFA.DIM1 + MFA.DIM2 | 135.87 | Full model |
|  | APPROACH ~ GENDER + MFA.DIM1 + MFA.DIM2 | 131.21 | AGE |
|  | APPROACH ~ MFA.DIM1 + MFA.DIM2 | 126.76 | GENDER |
|  | APPROACH ~ MFA.DIM1 | 125.11 | MFA.DIM2 |
