# Supplementary material for: Stakeholders’ perceptions of protected area management following a nationwide community-based conservation reform
Source: PLoS One. 2019 Apr 24;14(4):e0215437. doi: 10.1371/journal.pone.0215437 (PMC6481814; doi:10.1371/journal.pone.0215437)
Supplement: S11 Table — (DOCX) [file pone.0215437.s011.docx]

Supporting information for: Stakeholders’ perceptions of protected area management following a nationwide community-based conservation reform

## Table S11. Model output showing estimates of the log odds of the most parsimonious models following backwards elimination minimizing the AICc criterion.

|  | **Type** | **Model term** | **Estimate** | **Std. Error** | **Z-value** | **P-value** |  |
| --- | --- | --- | --- | --- | --- | --- | --- |
| Attitudes and MFA | Ordinal regression | MFA.DIM1 | -1.2566 | 0.2201 | -5.71 | 0.0000 | *** |
|  |  | MFA.DIM2 | -0.293 | 0.204 | -1.436 | 0.151 |  |
|  |  |  |  |  |  |  |  |
|  |  |  |  |  |  |  |  |
|  |  |  |  |  |  |  |  |
| Attitudes and conservation approach | Ordinal regression | APPROACH (Nature for people) | 1.242 | 0.924 | 1.344 | 0.1788 |  |
|  |  | APPROACH (People and nature) | 1.57 | 0.469 | 3.347 | 0.0008 | *** |
|  |  |  |  |  |  |  |  |
| Conservation approach and MFA | Multinomial regression | Intercept: APPROACH (Nature for people) | -1.942 | 0.501 | -3.881 | 0.0001 | *** |
|  |  | MFA.DIM1: APPROACH (Nature for people) | -0.195 | 0.351 | -0.556 | 0.5758 |  |
|  |  | Intercept: APPROACH (People and nature) | -0.158 | 0.276 | -0.574 | 0.5662 |  |
|  |  | MFA.DIM1: APPROACH (People and nature) | -0.924 | 0.227 | -4.068 | 0.0000 | *** |
|  |  |  |  |  |  |  |  |
