# Supplementary material for: Stakeholders’ perceptions of protected area management following a nationwide community-based conservation reform
Source: PLoS One. 2019 Apr 24;14(4):e0215437. doi: 10.1371/journal.pone.0215437 (PMC6481814; doi:10.1371/journal.pone.0215437)
Supplement: S12 Table — (DOCX) [file pone.0215437.s012.docx]

Supporting information for: Stakeholders’ perceptions of protected area management following a nationwide community-based conservation reform

Table S12. Management priorities by interest group. The percentage of participants that selected the various management priorities. Priorities selected by 50% or more of the participants within the different stakeholder groups are marked in blue. The number of participants within each interest group is noted in parenthesis.

|  | **Reduce land development** | **Stabilize land development** | **Traditional recreation** | **Novel recreation** | **Reduce traffic** | **Grazing & hay making** | **Modern farming** | **Commercial tourism** | **Nature-based tourism** | **Increase biodiversity** | **Maintain biodiversity** | **Access for disabled people** | **Reindeer herding** | **Cultural heritage** | **Industry & conservation** | **Property owners' interests** |
| --- | --- | --- | --- | --- | --- | --- | --- | --- | --- | --- | --- | --- | --- | --- | --- | --- |
| **Property owners (n=36)** | 3 | 19 | 28 | 3 | 31 | 67 | 19 | 3 | 25 | 3 | 31 | 11 | 8 | 33 | 3 | 44 |
| **Hunting and fishing (n=22)** | 9 | 45 | 32 | 0 | 45 | 59 | 23 | 0 | 27 | 9 | 41 | 5 | 0 | 50 | 5 | 32 |
| **Livestock (n=17)** | 0 | 29 | 41 | 0 | 53 | 71 | 18 | 0 | 18 | 0 | 41 | 6 | 6 | 47 | 6 | 35 |
| **Tourism (n=14)** | 0 | 21 | 36 | 14 | 43 | 50 | 14 | 7 | 43 | 14 | 21 | 7 | 0 | 29 | 7 | 7 |
| **Recreation (n=23)** | 22 | 35 | 39 | 4 | 48 | 52 | 22 | 0 | 30 | 30 | 35 | 9 | 0 | 43 | 4 | 26 |
| **Industry (n= 7)** | 0 | 0 | 0 | 14 | 29 | 43 | 29 | 0 | 14 | 29 | 71 | 0 | 0 | 43 | 29 | 29 |
| **Public administration (n= 11)** | 9 | 27 | 55 | 9 | 27 | 36 | 9 | 0 | 36 | 0 | 45 | 9 | 0 | 18 | 9 | 0 |
| **Cultural heritage (n=9)** | 22 | 33 | 11 | 0 | 33 | 67 | 0 | 0 | 22 | 11 | 44 | 0 | 11 | 33 | 0 | 22 |
| **Nature conservation (n= 16)** | 38 | 50 | 25 | 0 | 50 | 19 | 0 | 0 | 19 | 50 | 25 | 0 | 0 | 6 | 0 | 6 |
