# Supplementary material for: Stakeholders’ perceptions of protected area management following a nationwide community-based conservation reform
Source: PLoS One. 2019 Apr 24;14(4):e0215437. doi: 10.1371/journal.pone.0215437 (PMC6481814; doi:10.1371/journal.pone.0215437)
Supplement: S1 Fig — (DOCX) [file pone.0215437.s014.docx]

Supporting information for: Stakeholders’ perceptions of protected area management following a nationwide community-based conservation reform


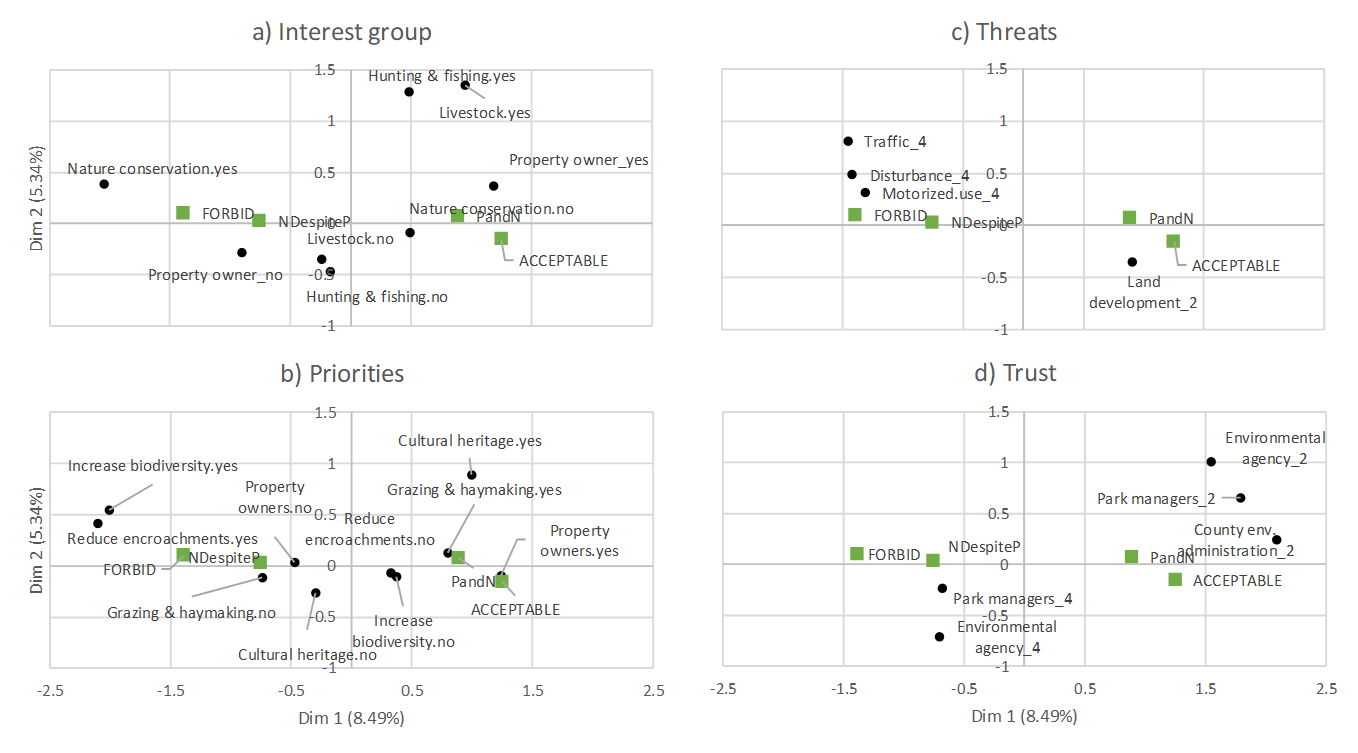


## Figure S1. A multiple factor analysis similar to the one in the main manuscript but with trust and threats as categorical variables. In figures a and b the indication of yes or no following the variable name indicates participants who selected this variable and those who did not. In figures c and d the numbers following the name of the variables (e.g. Traffic_4) shows the level of trust/threat from 1 = very low, 2 = low, 3= neither high or low/moderate, 4= high and 5= very high. The figures only shows variables that are well represented by the dimensions (a cos^2^ equal to or higher than 0.5) and that are important in accounting for the variation of the dimensions (i.e., contribute more than expected if the contribution of every variable was uniform).
