# Supplementary material for: Stakeholders’ perceptions of protected area management following a nationwide community-based conservation reform
Source: PLoS One. 2019 Apr 24;14(4):e0215437. doi: 10.1371/journal.pone.0215437 (PMC6481814; doi:10.1371/journal.pone.0215437)
Supplement: S1 Text — (DOCX) [file pone.0215437.s015.docx]

Supporting information for: Stakeholders’ perceptions of protected area management following a nationwide community-based conservation reform

## S1 text. Regression models of the associations between conservation approaches, attitudes and MFA dimensions.

We tested the relationship between attitudes towards PA loss or degradation and the resulting MFA dimensions, and the relationship between attitudes and conservation approaches, using ordinal regression models from the package ordinal in R [1,2]. Attitudes towards PA loss or degradation was an ordinal variable from 1-3, where 1 was forbid, 2 partly accept and 3 acceptable. We tested the relationship between conservation approaches and the two MFA dimensions using multinomial regression models from the package nnet [3]. In all the models we controlled for participant demographics (age and gender). Because only two participants supported the “nature for itself” approach, these were removed from the analyses along with those who did not have an opinion. Model selection was performed by minimizing the AICc.

Results

The most parsimonious model for the relationship between attitudes and MFA dimensions was ATTITUDES ~ MFA.DIM1 + MFA.DIM2. Age and gender were removed. There was a highly significant negative relationship between dimension one and acceptability towards PA loss or degradation (-1.26, SE = 0.22, P < 0.000), i.e., participants who accepted PA loss or degradation had low scores of dimension one. Dimension two was not significant (-0.29, SE = 0.20, P = 0.151). The most parsimonious model for the relationship between PA loss or degradation and conservation approach was ATTITUDES ~ APPROACH. Age and gender were removed. The acceptance of PA loss or degradation was significantly higher for participants who preferred the people and nature conservation approach (1.57, SE = 0.469, P = 0.008) compared with the reference level nature despite people. The difference between the reference level and nature for people was not significant (1.242, SE = 0.92, P = 0.179). The most parsimonious model for the relationship between conservation approach and MFA was APPROACH ~ MFA.DIM1. Age, gender and MFA.DIM2 were removed (see tables S10 and S11 for model selection and model output for the three models). The log odds that the participants preferred the people and nature conservation approach decreased with increasing values of MFA.DIM1 (-0.924, SE = 0.28, P <0.000).

# References

1. R Development Core Team. R: A language and environment for statistical computing. R Foundation for Statistical Computing, Vienna, Austria.; 2016.

2. Christensen RHB. Regression Models for Ordinal Data. Package ordinal. An R package. 2015; 1–22. Available: https://cran.r-project.org/web/packages/ordinal/ordinal.pdf

3. Ripley B, Venables W. Package “nnet.” 2016; 11.
