# Supplementary material for: Stakeholders’ perceptions of protected area management following a nationwide community-based conservation reform
Source: PLoS One. 2019 Apr 24;14(4):e0215437. doi: 10.1371/journal.pone.0215437 (PMC6481814; doi:10.1371/journal.pone.0215437)
Supplement: S2 Text — (DOCX) [file pone.0215437.s016.docx]

Supporting information for: Stakeholders’ perceptions of protected area management following a nationwide community-based conservation reform

## S12 Text:

## Questions in the questionnaire included in the study in English (in Norwegian further down), in the order they occurred.

**Which advisory council are you a member of? (Check on alternative**)

|  | **Midtre - Nordland** |
| --- | --- |
|  | **Breheimen** |
|  | **Dovrefjell** |
|  | **Jostedalsbreen** |
|  | **Jotunheimen** |
|  | **Naustdal-Gjengedal** |
|  | **Nærøyfjorden** |
|  | **Reinheimen** |
|  | **Stølsheimen** |
|  | **Trollheimen** |
|  | **Ålfotbreen** |
|  | **Other (specify here: .)** |

**Which interests do you primarily represent on the advisory council?** (Check all that apply)

|  | Property owners |
| --- | --- |
|  | Hunting & fishing |
|  | Livestock grazing |
|  | Tourism |
|  | Outdoor recreation |
|  | Industry |
|  | Public authority |
|  | Cultural heritage |
|  | Nature conservation |
|  | Other (specify here: ) |

**How long have you been a member of the advisory council? (The approximate number of months in total. Take into account all the months you have been a member, both those served consecutively and also if you have been a member periods with gaps in between)**

NUMBER OF MONTHS

**Do you have experience from protected area management from other bodies than the advisory council? (circle the right answer)**

**YES NO**

**To what degree to you believe that the conservation values are threatened by the categories listed?** (place an X for the right level of threat- only one per category)

|  | To a very large degree | To a large degree | To some degree | To a small degree | Not at all | No opinion |
| --- | --- | --- | --- | --- | --- | --- |
| Human activity in the protected area buffer zone |  |  |  |  |  |  |
| Woodland expansion |  |  |  |  |  |  |
| Alien species |  |  |  |  |  |  |
| Climate change |  |  |  |  |  |  |
| Overharvesting of plants and animals |  |  |  |  |  |  |
| Pollution |  |  |  |  |  |  |
| Human encroachments |  |  |  |  |  |  |
| Motorized vehicle use |  |  |  |  |  |  |
| Traffic in vulnerable areas |  |  |  |  |  |  |
| Other (Specify here:    ) |  |  |  |  |  |  |

**Economic development sometimes leads to the degradation of conservation values or the loss of protected land. Which of the following statements are closest to your opinion?** (Chose one option)

|  | This is acceptable because local commercial development is necessary. |
| --- | --- |
|  | This should be forbidden because these are our most important nature areas. |
|  | This is only acceptable for projects of especially large public interest and if the damage is fully compensated. |
|  | No opinion. |

**There are different ways to address environmental problems. How do you believe environmental problems are approached most effectively?** (Chose one option)

|  | Human activity is kept outside protected areas and nature is allowed to develop without interference |
| --- | --- |
|  | Environmental condition and threats are monitored, and populations managed to avoid negative effects of human activity as much as possible. |
|  | The great diversity of benefits provided by nature which humans depend on should be mapped and the costs to society if we lose these benefits should be measured. |
|  | Nature should, to a greater extent, be viewed as shaped by human use and focus should be placed on the interrelationships between nature and culture. |
|  | No opinion. |

**What would be your main priorities if you were granted decision-making power over protected areas?** (Check three that apply)

|  | Reduce the extent of human encroachments (e.g., buildings, communication towers, buildings, roads, fences are removed and areas are restored to their natural state). |
| --- | --- |
|  | Ensure that the extent of human encroachments do not increase. |
|  | Facilitate increased use of the protected areas for traditional, small-scale recreation. |
|  | Facilitate increased use of the protected areas for «modern» recreation (such as, cycling, kiting, alpine skiing, rafting, dog racing etc). |
|  | Limit traffic in vulnerable and ecologically important areas. |
|  | Maintain traditional grazing and hay making. |
|  | Facilitate a more economically viable, modern agriculture. |
|  | Facilitate profitable tourism in conjunction with the protected areas (cruise boats, alpine centers, hotels). |
|  | Facilitate the development of small-scale environmentally friendly nature based tourism. |
|  | Increase biodiversity in the protected areas using sound management so that the landscape to a greater degree is characterized as pristine nature/wilderness. |
|  | Ensure no loss of biodiversity in the protected areas. |
|  | Ease access to the protected areas for less mobile people (e.g. disabled and elderly people; e.g. through motorized transport or technical installations). |
|  | Facilitate greater room to maneuver for reindeer herding. |
|  | Ensure the protection of cultural heritage. |
|  | Balance economical development (e.g., mineral prospecting, energy production) along with the considerations for conservation values. |
|  | Ensure that property owners and other rights holders to a small degree are inconvenienced due to conservation restrictions. |
|  | Other (specify here ) |

**What is your level of trust in these protected area governance actors?** (Cross out for level of trust – one for each category)

|  | Very high trust | High trust | Neither high nor low trust | Low trust | Very low trust | No opinion |
| --- | --- | --- | --- | --- | --- | --- |
| Municipality |  |  |  |  |  |  |
| Local protected area boards |  |  |  |  |  |  |
| Secretariat  (park managers) |  |  |  |  |  |  |
| County Governor |  |  |  |  |  |  |
| Environmental Agency |  |  |  |  |  |  |
| The Ministry of Climate and Environment |  |  |  |  |  |  |
| The other members of the advisory council |  |  |  |  |  |  |

**We would like you to fill out some personal information below (These are only meant for registration and analyses. The study is completely anonymous.**

**Name .**

**Year of birth YEAR**

**Gender (circle the correct text)**

**WOMAN MAN**

**Highest education (chose one alternative)**

|  | **Elementary school/lower secondary school** |
| --- | --- |
|  | **High School** |
|  | **University/college** |

1. **E-post adresse**

**How did you find the survey?**

|  | **Easy and understandable** |
| --- | --- |
|  | **Damanding and understandable** |
|  | **Demanding and difficult to understand** |
|  | **Other (specify in the commentary field)** |

**General comments:**

## Opening page of the survey and consent form (in Norwegian).


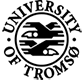


Hvilken rolle har de Rådgivende Utvalgene i forvaltningen?

I denne undersøkelsen fokuserer vi på betydningen av Rådgivende Utvalg (RU) for den lokale forvaltning av norske verneområder. Vi er interesserte i den kunnskapen som det rådgivende utvalget tilfører verneområdeforvaltninga, og hvordan denne bidrar til å oppnå verneformålene. Prosjektet er finansiert av Norges Forskningsråd og forskningen er ledet av UiT- Norges Arktiske Universitet.

Undersøkelsen består i et spørreskjema som fokuserer på kunnskaper, verdier og prioriteringer for forvaltning av verneområdene, samt noen spørsmål om den lokale forvaltningen.

Svarene dere bidrar med vil ikke kunne knyttes til enkeltpersoner, men vil inngå i generelle, overordnede analyser. NB! Husk å signere på samtykkeerklæringen før du sender inn skjemaet


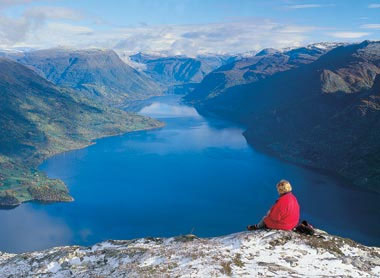


Prosjektpartnere
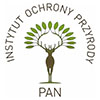

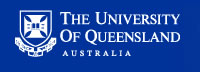


**Samtykke til deltakelse**

Vennligst les følgende informasjon før du bestemmer deg om du vil delta i dette studiet. Du bør være 18 år gammel eller eldre for å delta. Prosjektet har blitt godkjent av Norsk Samfunnsvitenskapelige Datatjeneste (www.nsd.uib.no, nr. 43238). Hvis du har noen etiske forbehold angående prosjektet eller spørsmål angående dine rettigheter som deltager, vennligst ta kontakt med nsd@nsd.uib.no.

**Formålet med studien**

UiT- Norges Arktiske Universitetet ønsker å undersøke betydningen av rådgivende utvalg for den lokale forvaltningen av norske verneområder. Resultatene vil forhåpentligvis gi økt kunnskap om involvering av ulike interessegrupper i forvaltningen

**Risiko og nytte**

Vi forutser ingen form for risiko ved å delta i denne studien. En mulig fordel for deg er at dine aktiviteter og preferanser blir identifisert og brukt i forvaltningen.

**Frivillig deltagelse**

Din deltakelse er fullstendig frivillig. Du kan trekke deg fra studiet når som helst. Vi vil på din oppfordring og ved framvisning av din tilgangskode fjerne dine svar fra våre databaser.

**Datainnsamling, lagring og bruk**

Resultatene vil lagres i en sikker database. Dine svar vil ikke kunne knyttes personlig til deg. All data vil bli sammenfattet og publisert i en rapport. Individuelle responser vil oppbevares av prosjektdeltakerne og vil ikke bli gjengitt i rapportering angående forskningen. Data som ikke er av identifiserbar karakter vil lagres i opptil 10 år.

**Rapportering av funn**

All lagret data som inneholder personlig informasjon vil forbli konfidensiell. Ingen informasjon som kan lede til identifikasjon av enkeltmennesker vil bli frigitt hvis ikke slikt kreves av lov. Et sammendrag av våre resultater vil legges ut på nettsiden og framtidige vitenskapelige publikasjoner vil være tilgjengelig på internettsiden til CultES (www.site.uit.no/cultes) og PPGIS-instituttet (www.landscapevalues.org).

**Kontaktinformasjon**

Ta gjerne kontakt med oss dersom du har noen spørsmål angående forskningen:

**Sigrid Engen (sigrid.m.engen@uit.no)**
**The Arctic University of Norway (Telephone: +47 776 44441)**

Dr. Vera Hausner (vera.hausner@uit.no)
The Arctic University of Norway (Telephone: +47 776 45905)

**Samtykkeerklæring**

Jeg har lest informasjonen angående denne forskningen og har fått tilfredsstillende svar på alle mine spørsmål. Jeg er 18 år eller eldre og samtykker frivillig til å delta. Jeg står fritt til å trekke meg fra studien på hvilket som helst tidspunkt. Jeg forstår at selv om informasjonen som blir samlet kan bli publisert så vil ikke jeg personlig kunne bli identifisert og mine personlige resultater vil forbli konfidensielle, hvis ikke annet kreves av lov.

UNDERSKRIFT DATO

## Questions from the questionnaire included in the study in Norwegian, in the order they occurred.

**Hvilket Rådgivende utvalg er du medlem av?** (Kryss av ett av alternativene)

|  | **Midtre - Nordland** |
| --- | --- |
|  | **Breheimen** |
|  | **Dovrefjell** |
|  | **Jostedalsbreen** |
|  | **Jotunheimen** |
|  | **Naustdal-Gjengedal** |
|  | **Nærøyfjorden** |
|  | **Reinheimen** |
|  | **Stølsheimen** |
|  | **Trollheimen** |
|  | **Ålfotbreen** |
|  | **Annet (spesifiser her: .)** |

**Hvilke interesser representerer du primært i utvalget?** (Kryss av alle aktuelle)

|  | Grunneiere |
| --- | --- |
|  | Jakt og Fiske |
|  | Beitenæringa |
|  | Turisme/reiseliv |
|  | Friluftsliv |
|  | Industri |
|  | Offentlig myndighet |
|  | Kulturminne |
|  | Miljøvern |
|  | Annet (spesifiser her: ) |

**Hvor lenge har du vært medlem av faglig rådgivende utvalg? (regn ut omtrentlig det totale antall måneder, selv om du har vært medlem over flere perioder med avbrudd i mellom)**

ANTALL MÅNEDER

**Har du erfaring fra verneområdeforvaltning fra andre organ enn faglig rådgivende utvalg? (sett ring rundt riktig svar)**

**JA NEI**

**I hvilken grad mener du at verneverdiene er truet av kategoriene nevnt nedenfor?** (kryss av for trusselnivå – ett kryss per bokstavkategori)

|  | I svært stor grad | I stor grad | I noen grad | I liten grad | Ikke i det hele tatt | Ikke grunnlag for å vurdere |
| --- | --- | --- | --- | --- | --- | --- |
| Menneskelig aktivitet i randsonen av verneområdene |  |  |  |  |  |  |
| Gjengroing |  |  |  |  |  |  |
| Fremmede arter |  |  |  |  |  |  |
| Klimaendringer |  |  |  |  |  |  |
| Overbeskatning av planter og dyr |  |  |  |  |  |  |
| Forurensning |  |  |  |  |  |  |
| Menneskelige inngrep |  |  |  |  |  |  |
| Motorisert ferdsel |  |  |  |  |  |  |
| Ferdsel i sårbare områder |  |  |  |  |  |  |
| Annet (Spesifiser her:    ) |  |  |  |  |  |  |

**Næringsutvikling fører noen ganger til at verneverdier forringes eller at vern av områder opphører. Hvilke av de følgende utsagnene er nærmest din oppfatning?** (Velg et alternativ – kryss av )

|  | Dette er akseptabelt fordi lokal næringsutvikling er nødvendig. |
| --- | --- |
|  | Dette bør forbys fordi disse er våre viktigste naturområder. |
|  | Dette er kun akseptabelt for prosjekter av særdeles stor offentlig interesse og dersom ødeleggelsene kompenseres fullt ut. |
|  | Ikke grunnlag for å vurdere. |

**Det finnes ulike måter å takle miljøutfordringer på. Hvordan mener du miljøutfordringer møtes mest effektivt?** (Velg ett alternativ – kryss av)

|  | Menneskelig aktivitet holdes utenfor vernede områder og naturen får utvikle seg fritt |
| --- | --- |
|  | Miljøtilstand og trusler overvåkes, og bestander forvaltes slik at man unngår negative effekter av menneskelig aktivitet mest mulig. |
|  | Man kartlegger det store spekteret av goder naturen leverer som mennesket er avhengig av, og setter søkelyset på hva det vil koste samfunnet hvis vi mister disse godene |
|  | Man må i større grad se på naturen som formet av menneskelig bruk og sørge for å ta vare på samspillet mellom natur og kultur. |
|  | Ikke grunnlag for å vurdere. |

**Hva ville vært dine hovedprioriteringer dersom du fikk ansvar for forvaltninga av vernede områder?** (sett kryss, men KUN tre stykker)

|  | Redusere omfanget av menneskelige inngrep (f.eks. bygninger, telemaster, veier, gjerder fjernes og områder tilbakeføres til naturen). |
| --- | --- |
|  | Sørge for at omfanget av menneskelige inngrep ikke øker |
|  | Tilrettelegge for økt bruk av verneområdene med tanke på tradisjonelt, enkelt, friluftsliv. |
|  | Tilrettelegge for økt bruk av verneområdene for «moderne» friluftslivsaktiviteter (f.eks. sykling, kiting, alpint, rafting, hundekjøring etc.). |
|  | Begrense ferdselen i sårbare og økologisk viktige områder. |
|  | Opprettholde tradisjonell beitebruk og slåtter. |
|  | Tilrettelegge for et mer økonomisk levedyktig, moderne beitebruk/landbruk. |
|  | Tilrettelegge for inntektsbringende turistvirksomhet i tilknytning til verneområdene (cruisebåttrafikk, alpinsenter, hotell). |
|  | Tilrettelegge for utviklingen av små-skala, miljøvennlig, naturbasert turisme. |
|  | Øke det biologiske mangfoldet i verneområdene ved hjelp av gode bevarings-/forvaltingsrettede tiltak slik at landskapet i større grad preges av urørt natur/villmark. |
|  | Sørge for at det biologiske mangfoldet i verneområdene ikke reduseres. |
|  | Tilrettelegge for at også ressurssvake grupper (f.eks. bevegelseshemmede og eldre) kan få oppleve verneområdene (persontransport og teknisk tilrettelegging for økt fremkommelighet). |
|  | Tilrettelegge for større handlingsrom for reindrift |
|  | Sikre bevaringen av kulturminner og kulturmiljø. |
|  | Balansere økonomisk utvikling (e.g. mineralutvinning, kraftproduksjon) med hensynet til verneverdier. |
|  | Sørge for at grunneiere og andre rettighetshavere i liten grad opplever belastning som følge av vernerestriksjoner. |
|  | Annet (spesifiser her ) |

**Hvor stor grad av tillit har du til disse aktørene innen verneområdeforvaltninga?** (Kryss av for nivå av tillit - ett kryss per bokstavkategori)

|  | Svært høy tillit | Høy tillit | Verken høy eller lav tillit | Liten tillit | Svært liten tillit | Ikke grunnlag for å vurdere |
| --- | --- | --- | --- | --- | --- | --- |
| Kommunen |  |  |  |  |  |  |
| Verneområdestyret |  |  |  |  |  |  |
| Sekretariatet (nasjonalparkforvaltere) |  |  |  |  |  |  |
| Fylkesmannens miljøvernavdeling |  |  |  |  |  |  |
| Miljødirektoratet |  |  |  |  |  |  |
| Klima og miljødepartementet |  |  |  |  |  |  |
| De andre medlemmene i faglig rådgivende utvalg |  |  |  |  |  |  |

**Nedenfor er det fint om du fyller ut noen personopplysninger (Disse er kun ment for registrering og analyser. Undersøkelsen er anonym).**

**Navn .**

**Fødselsår ÅRSTALL**

**Kjønn (sett ring rundt rett tekst)**

**KVINNE MANN**

**Høyeste utdanning (velg ett alternativ – sett kryss)**

|  | **Ungdomsskole/realskole** |
| --- | --- |
|  | **Videregående/yrkesfag** |
|  | **Universitet/høyskole** |

1. **E-post adresse**

**Skulle du ha tanker eller erfaringer til slutt som du mener kan bidra til å forbedre ordninga med faglig rådgivende utvalg, som ikke har fanget tilstrekkelig opp i spørsmålene ovenfor, vil vi gjerne at du deler disse med oss nedenfor.**

***Tusen takk for at du tok deg tid til å delta!***

**Hvordan opplevde du spørreundersøkelsen?**

|  | **Enkel og forståelig** |
| --- | --- |
|  | **Krevende og forståelig** |
|  | **Krevende og vanskelig å forstå** |
|  | **Annet (spesifiser i kommentarfelt)** |

**Generelle tilbakemeldinger/kommentarer:**
